# The α1,6-Fucosyltransferase Gene (*fut8*) from the *Sf*9 Lepidopteran Insect Cell Line: Insights into *fut8* Evolution

**DOI:** 10.1371/journal.pone.0110422

**Published:** 2014-10-21

**Authors:** Sylvie Juliant, Anne Harduin-Lepers, François Monjaret, Béatrice Catieau, Marie-Luce Violet, Pierre Cérutti, Annick Ozil, Martine Duonor-Cérutti

**Affiliations:** 1 CNRS UPS3044 Baculovirus et Thérapie, Saint Christol Lèz Alès, France; 2 CNRS UMR8576, Unité de Glycobiologie Structurale et Fonctionnelle, Université Lille Nord de France, Lille1, Villeneuve d'Ascq, France; 3 Laboratoire Français du Fractionnement et des Biotechnologies de Lille, Lille, France; Griffith University, Australia

## Abstract

The core alpha1,6-fucosyltransferase (FUT8) catalyzes the transfer of a fucosyl moiety from GDP-fucose to the innermost asparagine-linked *N*-acetylglucosamine residue of glycoproteins. In mammals, this glycosylation has an important function in many fundamental biological processes and although no essential role has been demonstrated yet in all animals, FUT8 amino acid (aa) sequence and FUT8 activity are very well conserved throughout the animal kingdom. We have cloned the cDNA and the complete gene encoding the FUT8 in the *Sf9* (*Spodoptera frugiperda*) lepidopteran cell line. As in most animal genomes, *fut8* is a single-copy gene organized in different exons. The open reading frame contains 12 exons, a characteristic that seems to be shared by all lepidopteran *fut8* genes. We chose to study the gene structure as a way to characterize the evolutionary relationships of the *fut8* genes in metazoans. Analysis of the intron-exon organization in 56 *fut8* orthologs allowed us to propose a model for *fut8* evolution in metazoans. The presence of a highly variable number of exons in metazoan *fut8* genes suggests a complex evolutionary history with many intron gain and loss events, particularly in arthropods, but not in chordata. Moreover, despite the high conservation of lepidoptera FUT8 sequences also in vertebrates and hymenoptera, the exon-intron organization of hymenoptera *fut8* genes is order-specific with no shared exons. This feature suggests that the observed intron losses and gains may be linked to evolutionary innovations, such as the appearance of new orders.

## Introduction

Glycosylation of proteins is a key process. Indeed, congenital disorders of glycosylation lead to severe dysfunction and disability. Maturation of glycoproteins in the Golgi apparatus requires hundreds of enzymes (i.e., glycosyltransferases, glycosidases), known as Carbohydrate-Active Enzymes (CAZymes) [1], and also chaperones that act through complex protein-protein interactions.

Fucosylation is one of the most common post-translational modifications. Fucosylated glycans are involved in various biological processes, such as cell adhesion, growth factor receptor modulation, microbial and viral infections, cancer and atherosclerosis [for review 2, 3]. Several fucosyltransferases (FucTs) have been identified and classified in the CAZy glycosyltransferase (GT) families GT-11 (α1,2-FucTs), GT-10 (α1,3/4-FucTs), GT-23 (core α1,6-FucTs, known as fut8), GT-37 (α1,2-FucTs), GT-65 (O-FucTs pofut1 and fut12) and GT-68 (O-FucTs pofut2 and fut13). A previous phylogenetic analysis of vertebrates, invertebrates and bacterial FucTs genes highlighted their ancient and divergent evolution from one or two ancestral genes [4].

While α1,2- and α1,3/4-FucTs are implicated in terminal fucosylation (e.g., histo-blood group antigens) [5], core α1,6-FucT (FUT8) adds fucose to the innermost asparagine-linked *N*-acetylglucosamine (GlcNAc) of the chitobiose disaccharide-core unit of glycoproteins. This core fucosylation has an essential role in regulating the function of many glycoproteins, such as activation of growth factor receptors [6] and modulation of antibody-dependent cell-mediated cytotoxicity (ADCC) *in vitro* and *in vivo* [7]. In mammals, FUT8 physiological relevance has been demonstrated by genetic ablation of the *fut8* gene: 80% of these mice die three days after birth [8] and the survivors present severe growth retardation.

The crystal structure of human FUT8 has been resolved [9]. It is a type-II glycoprotein with a short N-terminal cytoplasmic tail, a transmembrane domain, a stem region and a C-terminal catalytic domain. The latter encompasses three peptidic consensus sequences (motif I, motif II and motif III) [4], [9] located in the Rossmann fold [10], a protein motif that binds to nucleotides. These motifs are conserved among α1,2-, α1,6- and *O*-FucTs, strongly suggesting their implication in the fucose transfer reaction [4], [11]–[14]. Site-directed mutagenesis [9], [12] confirmed this hypothesis demonstrating that most of the highly conserved aa in this region are essential for enzymatic activity. Four disulfide bonds may be important for protein folding and stability [9]. A SH3 domain is also present at the C-terminus in all known FUT8 sequences [15], [16], but its role has not been elucidated yet.

Lepidopteran cells can perform most of the post-translational modifications, including *N*- and *O*-glycosylation. *N*-glycan processing [for review, 17] is comparable to what observed in mammalian cells during the early stages of the glycosylation pathway with the synthesis of GlcNAcβ1,2Manα1,3[Manα1,6]Manβ1,4GlcNAcβ1,4[Fucα1,6]GlcNAc [18]. The glycan structures most frequently identified on glycoproteins synthesized in insect cells are fucosylated paucimannose structures (Man_3_(Fuc)GlcNAc_2_) and, to a lesser extent, oligomannose-type glycans [19]. The absence of complex oligosaccharides correlates with the absence of β1,4-galactosyltransferase I (β1,4GalTI) [20], [21] and sialyltransferases [18]. Despite the *in silico* identification of α2,6-sialyltransferase (*st6gal*) gene sequences in some lepidopterans, such as *Bombyx mori* [22], no sialyltransferase activity has been detected in insect cell lines, such as *Sf*9 (*S. frugiperda*) cells [18], [23]. In addition, the very low expression level of *N*- acetylglucosaminyltransferase I (GNT-I) [18], [24], [25] and GNT-II [26] and the presence of a Golgi-associated *N*-acetylglucosaminidase (FDL) [27], [28] explain the formation of paucimannose structures. In contrast to mammals, two core-fucosylation events with α1,6- and α1,3-linkage have been reported in insects and in plants [29]. Analysis of fucosyltransferase activities in several lepidopteran cell lines showed distinct activities in each cell line. While core α1,3- and core α1,6-fucose are found on glycoproteins expressed in *Masmestra brassicae* [20], [30], [31] and *Trichoplusia ni* (High five) cell lines [32], only core α1,6-fucose was found in *B mori* and *Sf9* cells [20], [30], [33].

Although its essential role has not yet been demonstrated in all animals, FUT8 aa sequence and FUT8 enzymatic activity are well conserved throughout the animal kingdom as testified by several molecular cloning and functional studies in vertebrates and invertebrates [34], [35], [36], [37], [38]. In the present study, we report the molecular cloning and functional characterization of a cDNA encoding *fut*8 from the *Sf9* lepidopteran insect cell line. As in most animal genomes, *fut8* is a single-copy gene organized in several exons. These properties and the high conservation of amino acids in FUT8 catalytic domain were used to retrieve unique *fut8* orthologs from a large variety of metazoan genomes and to study their exon-intron structure evolution in several insect orders. The results of this analysis allow us to propose a model of *fut8* evolution throughout the animal kingdom. Furthermore, *fut8* evolutionary history could be used to measure the divergences among insect genomes highlighting the frequent intron losses and gains in arthropods.

## Results

### Cloning of *fut8* cDNA from *Sf9* cells

A partial cDNA sequence was obtained using a reverse transcription - polymerase chain reaction (RT-PCR) approach and degenerate primers (**Table S1**). This 860-bp (base pairs) sequence was compared to all non-redundant coding sequences (cds) in the GenBank database using the BlastX program [39] and was identified as belonging to a *fut8* gene, with a high identity score (51% aa identity with human FUT8). This sequence was then used to design new exact-match primers for 3′- and 5′-RACE. The full-length cDNA sequence of *Sf*9 *fut8* gene is available under the accession number KC538901.

**Figure 1:**
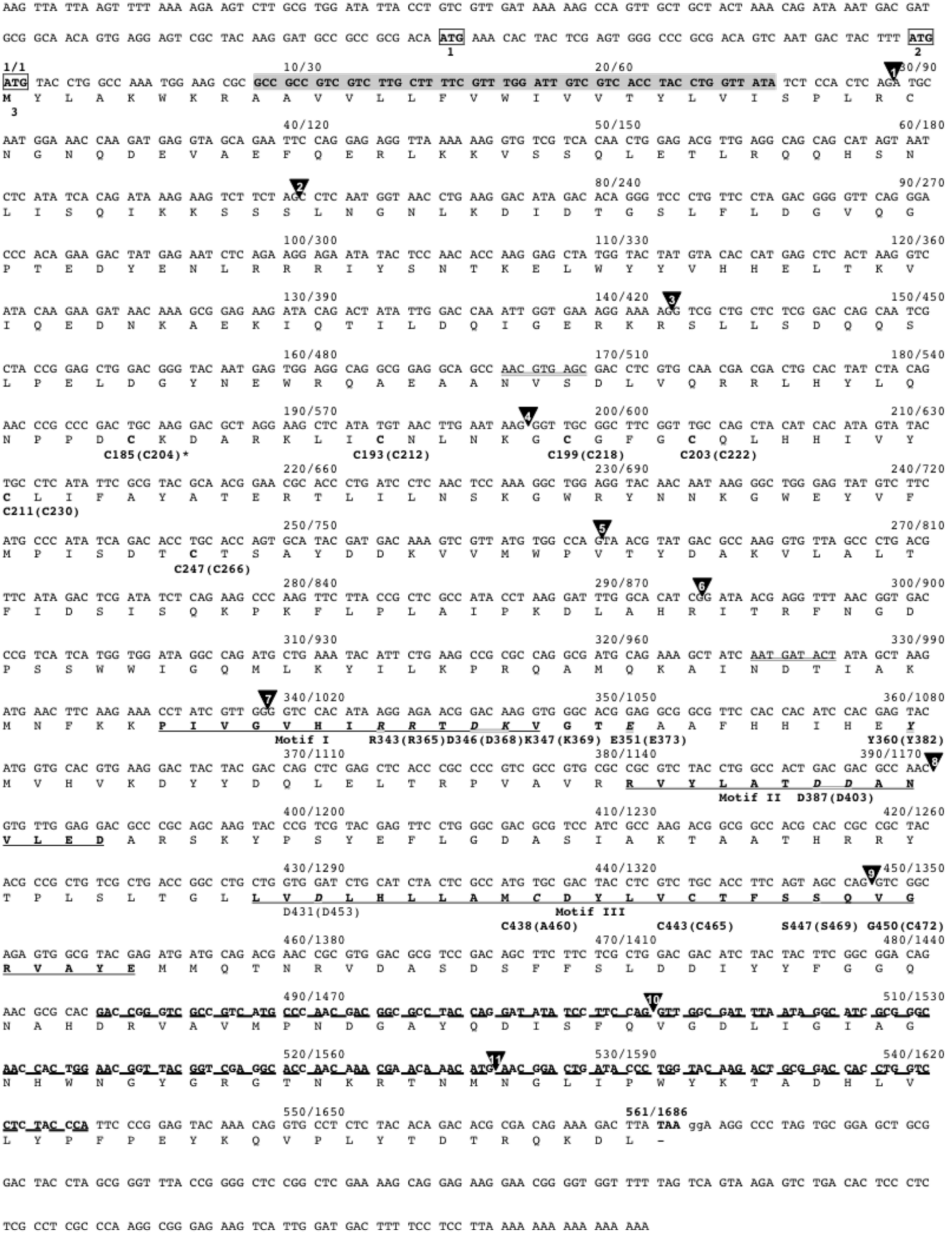
Nucleotide and deduced aa sequences of the *Sf*9 α1,6-fucosyltransferase cDNA. The three potential start codons are presented in boxes. ATG3 was considered as the start codon (see Table 2). A potential transmembrane domain (residues 9 to 25) is highlighted in grey. The conserved motifs I, II and III in the active site are underlined. The putative SH3 domain is in bold and underlined with a dashed line. The two potential *N*-glycosylation sites are underlined with a double line. Solid arrowheads indicate the position of introns. The numbers between brackets represent the position of critical aa conserved in human FUT8.

Sequence analysis of this full-length cDNA sequence showed the presence of three in-frame ATG codons as potential initiation codons (**Figure 1**). Inspection of the sequences immediately flanking the initiation codon of genes expressed in *Sf*9 cells (n = 168) [40] allowed us to propose a consensus sequence (**Table 1**) for initiation of translation (most frequent for gene expression with high or low efficiency) that was in agreement with the start translation sites reported by Cavener and Ray [41] for invertebrates. Based on this consensus sequence, the third ATG codon (**Figure 1**, ATG3) seemed to have the most favorable environment as potential initiation codon (**Table 2**). Sequence analysis showed that this cDNA encodes a type II protein of 561-aa with a short cytoplasmic tail of 8-aa at the N-terminus followed by a 17-aa (residues 9–25) transmembrane domain. The Golgi luminal part contains the catalytic C-terminal domain with the three highly conserved motif I (residues 336–348), motif II (residues 381–394) and motif III (residues 429–455) consensus sequences [4], [9]. All the critical aa residues (Arg-343, Asp-346, Lys-347, Glu-351, Tyr-360, Asp-387, Asp-431 and Ser-447) implicated in FUT8 enzymatic activity are also perfectly conserved in the *Sf9* Fut8 protein (**Figure 1**). The catalytic domain is preceded by a 78-aa proline-rich region (Pro-279 to Pro-336), like in mammalian FUT8 [15], [35], [36]. As for all known FUT8 sequences, *Sf9* FUT8 has a 60-aa SH3 domain at its C-terminus (residues 484–543) [15], [16] (**Figure 1**). All cysteine residues involved in the formation of disulfide bridges in human FUT8, are also perfectly conserved, except Cys-472 in motif III that is replaced by a glycine residue (G-450) (**Figure 1** **and Figure S1**). The amino acid identity of the *Sf9* FUT8 aa sequences was 86.81%, and 79.89% with the lepidopteran *Manduca sexta* and *Danaus Plexippus* FUT8. Identity with dipteran FUT8 sequences ranged between 50.28% (*Culex quinquefasciatus pipiens)* and 46.90% (*Drosophila melanogaster)* and was about 44% with vertebrate FUT8 aa sequences (44.24% with human and 44.42% with bovine *fut8*).

**Table 1:** Determination of the optimal sequence for initiation of translation in *Sf*9 cells.

Sequences analyzed n = 168	Position upstream of the start codon*										
	-10	-9	-8	-7	-6	-5	-4	-3	-2	-1	AUG
**A**	**43**	**36**	**27**	**35**	**27**	22	**39**	**88**	**64**	**57**	
**U**	26	**34**	**29**	**27**	**31**	**41**	11	0	13	6	
**G**	14	17	14	7	**28**	12	7	10	3	11	
**C**	17	13	**30**	**31**	14	25	**43**	2	20	26	
**Consensus sequence**	**A**	A/U	A/U/C	A/U/C	A/U/G	**U**	A/C	**A**	**A**	**A**	**AUG**

168 genes expressed in *Sf*9 cells [40] were included in this analysis.

*Nucleotide flanking *Sf*9 start codons, in percentage.

**Table 2:** Analysis of the sequences immediately flanking the three potential start codons (ATG1, ATG2 and ATG3 in Figure 1) identified in the *fut8* cDNA.

	Position upstream of the start codon										
	-10	-9	-8	-7	-6	-5	-4	-3	-2	-1	AUG
*Consensus sequence* 1	**A**	**A/U**	**A/U/C**	**A/U/C**	**A/U/G**	**U**	**A/C**	**A**	**A**	**A**	**AUG**
*Consensus sequence* 2	**A**	**U**	**A**	**A**	**A**	**U**	**A/C**	**A**	**A**	**C**	**AUG**
	C	G	**C**	**C**	**G**	C	G	**A**	C	**A**	**AUG (1)**
	U	G	**A**	**C**	**U**	A	**C**	U	U	U	**AUG (2)**
	C	**U**	**A**	**C**	**U**	**U**	U	**A**	U	G	**AUG (3)**

1 Consensus sequence determined in this work (Table 1).

2 Consensus sequence determined by Cavener and Ray (1991) [41] for invertebrates.

To confirm that this cDNA encoded *Sf9* FUT8, we evaluated the enzymatic activity of a recombinant protein produced by expressing the cloned cDNA in the baculovirus-*Sf9* cell expression system. As shown in **Table 3**, a characteristic fucosyltransferase activity was observed.

**Table 3:** Determination of α1–6-fucosyltransferase activity.

Sample	α1,6-fucosyltransferase activity
2D production	1,229+/−80
3D production	2,554+/−35
Negative control	62+/−1

Recombinant FLAG-tagged *Sf*9 FUT8 was produced using the baculovirus-*Sf*9 cell system and purified using anti-FLAG M2 affinity gel. The fucosyltransferase activity of the recombinant protein was tested with GDP-[^14^C]-L-fucose, as substrate, after two days (2D) and three days (3D) of production. The transfer of [^14^C]-fucose is expressed in cpm.

**Figure 2:**
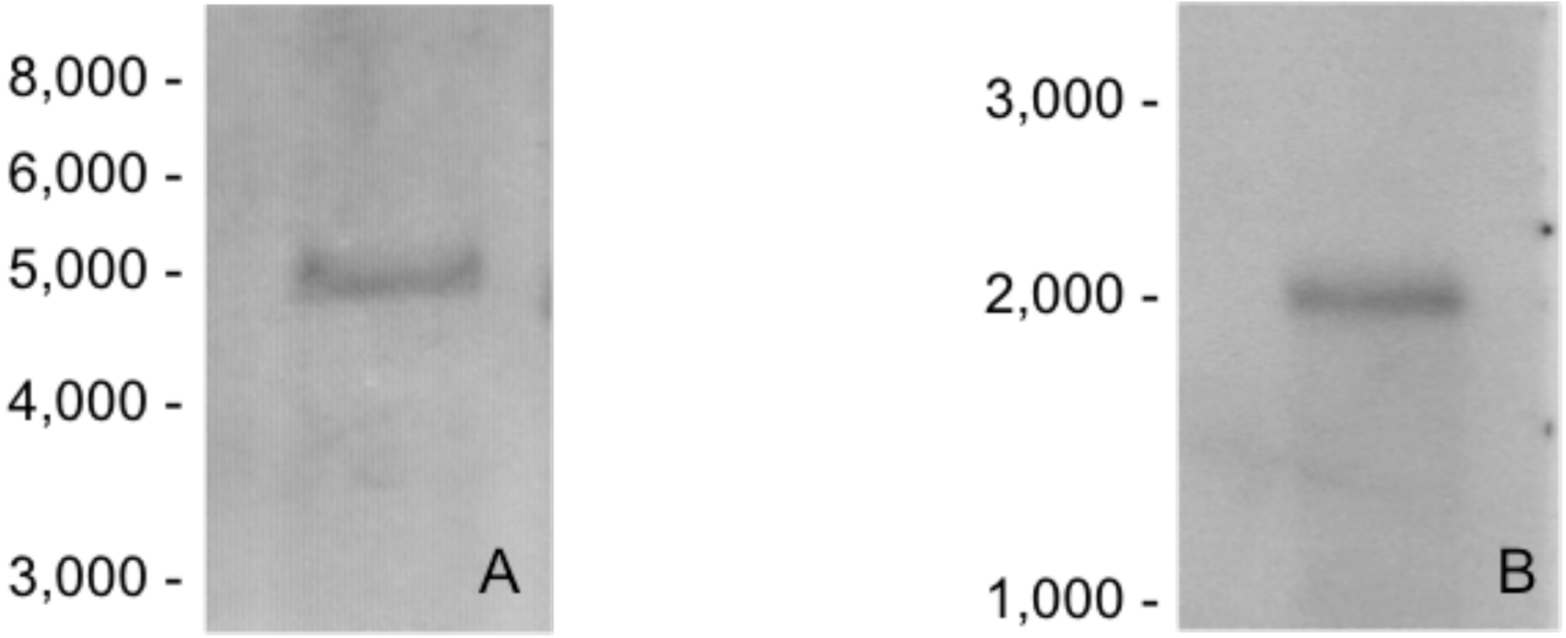
Southern and Northern blot analyses. **A.** Ten µg/lane of EcoRI-digested genomic DNA from *Sf*9 cells was hybridized with a mono-exonic (exon 3, {E*3*l}) digoxigenin-labeled Fut8 probe prepared by PCR using the Forint10 and B24 primers (Table S1), as described in Materials and Methods. **B.** One µg/lane of total RNA was analyzed by Northern blotting. Hybridization was performed with a *fut8* anti-sense RNA probe (pos. 825 to pos. 1686 on the cDNA, Figure 1). Molecular weight markers in bp are indicated on the left of each panel.

Restriction-enzyme digested genomic DNA from *Sf*9 cells hybridized to a single fragment, suggesting the presence of only one copy of the *fut8* gene in the *Sf*9 genome (**Figure 2A**). *Fut8* expression was then analyzed by Northern blotting using a specific mono-exonic (exon 3) *fut8* probe. Only one transcript of the expected size was detected (**Figure 2B**).

### Phylogenomic analyses

**Figure 3:**
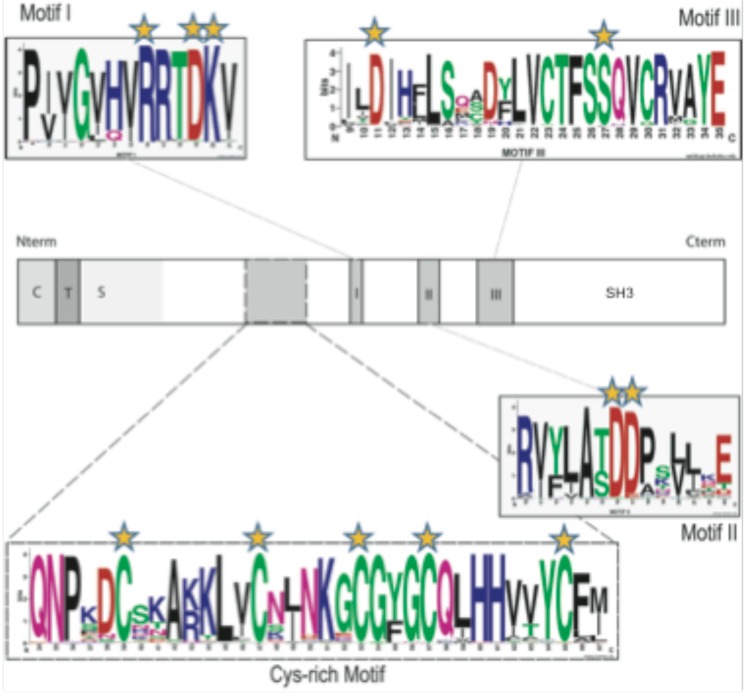
Conserved aa and motifs found in all the α1,6-fucosyltransferases sequences. Schematic representations of the FUT8 protein showing the cytoplasmic (C), transmembrane (T) and stem region (S) characteristic of α1,6-fucosyltransferases. The catalytic domain is in white and motifs I, II and III in grey. In addition, a region found only in α1,6-fucosyltransferase with conserved cysteine residues is indicated by dashed lines and was named “Cys-rich” domain. Conserved aa and those implicated in the enzymatic activity are highlighted with orange stars. The conserved peptide sequences used to generate the motif I, motif II and motif III sequence logos were extracted from multiple alignments of 96 α1,6-fucosyltransferase sequences identified in the databases (Table S2) and visualized at the Weblogos site at Berkeley, as described previously [63]. In the logos, aa are colored according to their chemical properties: polar aa (G, C, S, T, Y) are green, basic (K, R, H) are blue, acidic (D, E) are red, hydrophobic (A, V, L, I, P, W, F, M) are black and neutral polar aa (N, Q) are pink. The overall height of the stacks indicates the sequence conservation at a given position, while the height of the symbol within the stack indicates the relative frequency of each aa at that position. [69], [70].

We took advantage of the growing list of sequenced metazoan genomes to identify *in silico* several potential orthologs of the previously described FUT8 protein sequences. BLAST searches using the human FUT8 sequence and the previously described [4], [9] fucosyl motifs I, II and III, as hallmarks for ortholog identification, allowed the identification of 96 highly conserved FUT8-related sequences from early metazoan genomes to protostome (mainly arthropods) and deuterostome genomes (**Table S2**). In most of these genomes, one single-copy *fut8* gene was identified with the notable exception of *Danio rerio, Xenopus laevis* and *Saccoglossus kowalevskii* in which two *fut8* paralogs (named fut8A and fut8B) were found. Multiple sequence alignments using ClustalW (**Figure 3**) highlighted the presence of the three conserved motifs (motifs I, II and III) and of a new “Cys-rich” peptide motif that is highly specific to FUT8 proteins.

**Figure 4:**
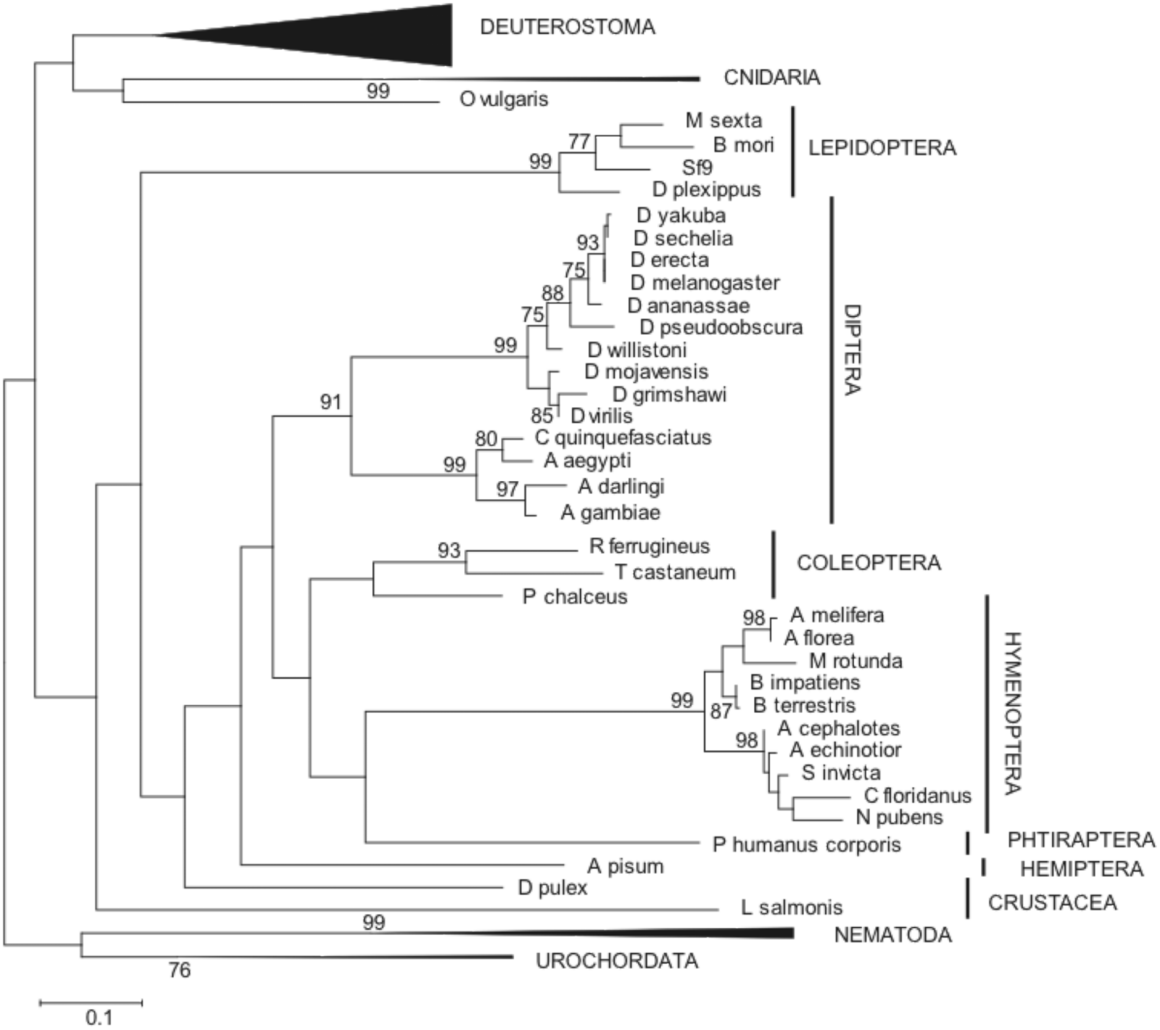
Molecular phylogenetic analysis using the Maximum Likelihood method. Evolutionary analyses were conducted in MEGA5 [67] and the evolutionary history was inferred using the Maximum Likelihood method based on the Whelan and Goldman model [72]. This analysis involved 92 FUT8 aa sequences and the final dataset contained 336 positions (42% of 787). The bootstrap consensus tree inferred from 1050 replicates is taken to represent the evolutionary history of the analyzed taxa. The percentage of trees (only those >75%) in which the associated taxa clustered together is shown next to the branches [73]. Initial tree(s) for the heuristic search were obtained automatically as follows. When the number of common sites was <100 or less than one fourth of the total number of sites, the maximum parsimony method was used; otherwise the BIONJ method with the MCL distance matrix was used.

We then assessed the evolutionary relationships of these animal sequences as described in the Materials and Methods section. To understand the orthology relationships of arthropods FUT8 sequences, we performed Maximum Likelihood (ML) (**Figure 4**) and Neighbor Joining (NJ) reconstructions (**Figure S2**). The recovered ML topology was very similar to the one generated by the NJ method, showing high conservation in vertebrates and in each insect order. The newly described *S. frugiperda fut8* sequence grouped with other early diverging lepidopteran FUT8 sequences. Both tree topologies tended to place nematode FUT8 sequences outside the protostome branch suggesting that these are rapidly evolving FUT8 sequences. We also investigated the extent of synteny and gene order conservation in the metazoan FUT8 sequences visualized at the Genomicus web site [42]. This preliminary analysis suggested microsynteny conservation in each insect order and no synteny conservation between insect orders (data not shown).

### Intron-exon organization of the *fut8* genes

**Figure 5:**
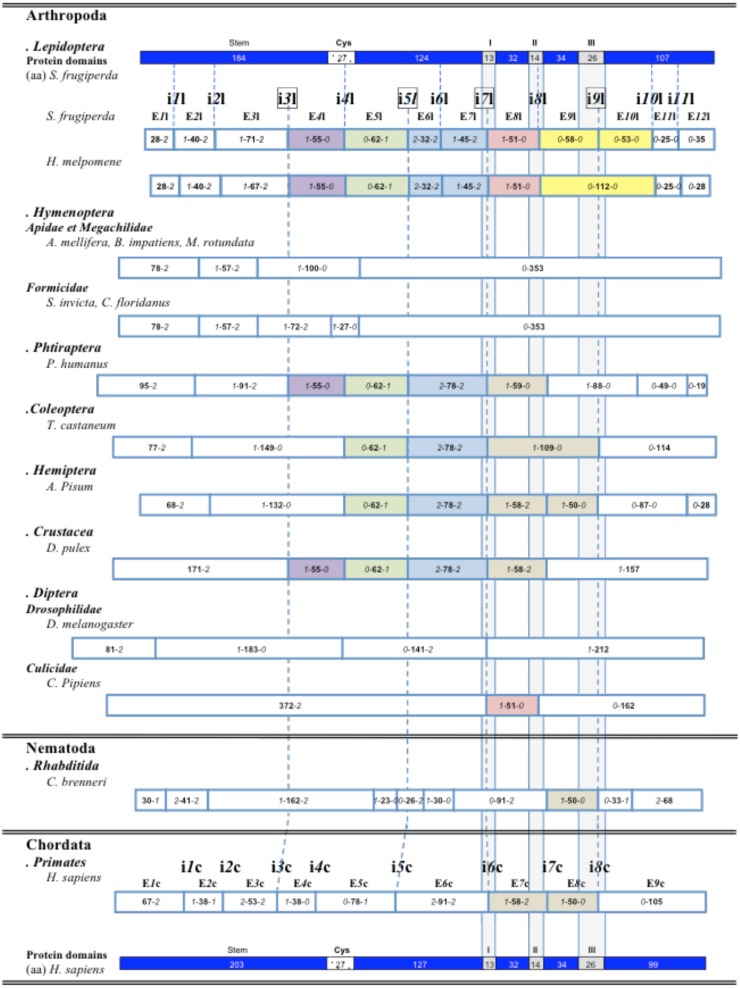
Schematic illustration of the genomic organization of the *fut8* genes identified in animal genomes. Coding exons are represented by rectangles with their relative size in codons (in bold). Italic numbers represent the number of nucleotides belonging to codons that flank the intron insertion site and determine the intron insertion phase. Dashed lines represent conserved intron insertion sites. Conserved exons are in the same color. *S. frugiperda* and *H. sapiens* FUT8 proteins are in blue, specific domains are represented by rectangles with their relative size in aa (Cys-rich domain, in black and motifs I, II and III, in grey).

Twelve exons were identified in *Sf9 fut8,* ranging from 75 bp to 216 bp in size. The intron-exon boundaries of *Sf*9 *fut8* were in agreement with the consensus sequence for the splicing donor and acceptor sites (GT at position 1 and 2 at 5′ splice sites and AG at position -1 and -2 at 3′ splice sites) (not shown) [43], [44]. Comparative analysis of *fut8* orthologs showed that the intron-exon organization was order-specific (**Figure 5**). The structure of lepidopteran *fut8* genes was overall well-conserved with only minor length disparities in exons 3, 9 and 12. Particularly, exon 3 contained 67 codons in *Heliconius melpomene*, 69 in *D. plexippus* and 71 codons in *S. frugiperda* and *B. mori*; exon 9 of *H. melpomene fut8* included 112 codons as a result of exon 9 and 10 fusion.

Despite the overall highly variable organization of *fut*8 genes (**Figure 5**), some exons (such as exons 4 and 8) that encode domains involved in protein folding and catalytic activity were shared among arthropod *fut8* genes. Exon 4 was also present in *Pediculus humanus* (phtiraptera) and *Daphnia pulex* (diplostraca) *fut8*, whereas exon 5 was found in *P. humanus, Tribolium castaneum* (coleoptera), *Acyrthosiphon pisum* and *D. pulex fut8*. Exon 8 was also found in Culicidae (e.g., *C. pipiens and Anopheles gambiae*). Lepidopteran exons 6 and 7 seemed to result from the splitting of an exon shared (*2-*78-*2*) by many arthropoda (phtiraptera, coleoptera, hemiptera and crustacea).

*Sf*9 *fut8* contained 11 introns (noted i*1*l to i*11*l), a much higher number compared to other insect *fut8* genes (between 2 and 9 introns) (**Table 4**). Diptera had the lowest number of introns: 3 in Drosophilidae and 2 in Culicidae. Strikingly, although all the essential sequence motifs required for the enzymatic activity were conserved in the hymenoptera *fut8* genes, the intron insertion sites identified in these genes (three in *Apis mellifera*, *Bombus impatiens, Megachile rotundata* and four in *Atta cephalotes, Solenopsis invicta, Camponotus floridanus, Harpagnathos saltator*) were hymenoptera-specific and were not shared by any of the other arthropod f*ut8* genes analyzed here. In addition, the intron position i*3*h, which splits the hymenopteran exon 3 in apidae and megachilidae, appeared to be formicidae-specific (**Table 5** **,** **Figure 5**).

**Table 4:** Analysis of intron positions shared with *S. frugiperda fut8* (i*number*l) in arthropoda and chordata orthologs *fut8* genes.

Protein domain				Stem			Cys-rich			Motif I	Motif II	Motif III			Total IS (bp)	Nb introns
Intron insertion sites				i*1*l	i*2*l	i*3*l	i*4*l	i*5*l	i*6*l	i*7*l	i*8*l	i*9*l	i*10*l	i*11*l		
**Arthropoda**	**Insecta**	Lepidoptera	*S. frugiperda*	**2/3,459**	**2/344**	**2/1,645**	**0/8,467**	**1/3,290**	**2/733**	**2/1,097**	**0/989**	**0/494**	**0/601**	**0/952**	22,071	11
			*B. mori*	**2/3,280**	**2/2,841**	**2/5,599**	**0/1,203**	**1/1,197**	**2/355**	**2/483**	**0/1,167**	**0/1,971**	**0/1,188**	**0/795**	20,079	11
			*D. plexippus*	**2/4,756**	**2/312**	**2/1,263**	**0/2,173**	**1/512**	**2/299**	**2/1,177**	**0/1,393**	**0/468**	**0/590**	**0/71**	13,014	11
			*H. melpomene*	**2/1,633**	**2/712**	**2/1,821**	**0/7,380**	**1/1111**	**2/204**	**2/146**	**0/795**		**0/129**	**0/248**	14,179	10
		Coleoptera	*T. castaneum*				**0/9,139**	**1/52**		**2/1,524**		**0/8,806**			21,186	5
		Hemiptera	*A. pisum*				**0/67**	**1/91**		**2/118**		**0/62**			752	7
		Phtiraptera	*P. humanus*			**2/342**	**0/82**	**1/215**		**2/295**					3,507	9
		Diptera	*D. melanogaster*				**0/109**			**2/65**					582	3
			*C. pipiens*							**2/62**	**0/98**				160	2
			L. longipalpis*							**2/94**	**0/438**				≥3,290	≥3
	**Arachnida**	Mesostigmata	*M. occidentallis*									**0/210**			1,581	8
		Trombidiformes	*T. urticae*												1,173	6
	**Branchiopoda**	Cladocera	*D. pulex*			**2/65**	**0/60**	**1/61**		**2/55**					320	5
	**Maxilopoda**	Siphonostomatoida	*L. salmonis*												0	0
	**Chilopoda**	Geoplilomorph	*S. maritima*							**2/292**		**0/56**			2,591	6
**Chordata**	**Acidiacae**	Enterogona	*C. intestinalis A*			**2/836**		**1/906**		**2/820**		**0/447**	**0/5,797**		10,989	10
	**Mammalia**	Primate	*H. sapiens*			**2/13,118**		**1/52,295**		**2/2,125**		**0/8,711**			130,359	8
		Rodentia	*R. norvegicus*			**2/19,848**		**1/36,598**		**2/1,403**		**0/11,243**			131,971	8
		Artiodactyla	*B. taurus*			**2/14,139**		**1/40,145**		**2/4,795**		**0/11,832**			173,134	8

IS: intronic sequence, Nb: number. The intron phase was defined as follows: phase 0 introns are inserted between two codons; phase 1 introns are inserted after the first nucleotide of the codon, and phase 2 introns are inserted between the second and the third nucleotide of the codon.

(*) The gene is truncated and the 5′ exon is missing.

**Table 5:** Analysis of intron positions in *fut8* orthologs in hymenoptera (i*number*h).

Protein domain				Stem		Cys-rich			
Intron insertion sites				i*1*h	i*2*h	i*3*h	i*4*h	Total IS (bp)	Nb introns
**Insecta**	**Hymenoptera**	Apidae	*A. mellifera*	**2/49**	**2/85**		**0/109**	243	3
			*B. impatiens*	**2/101**	**2/84**		**0/84**	269	3
		Megachilidae	*M. rotundata*	**2/101**	**2/75**		**0/65**	241	3
		Formicidae	*A cephalotes*	*0/92*	**2/80**	**2/96**	**0/84**	352	4
			*S. invicta*	**2/725**	**2/84**	**2/130**	**0/71**	1010	4
			*C. floridanus*	**2/116**	**2/77**	**2/137**	**0/75**	405	4
			*H. saltator*	**2/145**	**2/89**	**2/97**	**0/271**	457	4

The intron phase was defined as described in Table 4. IS: intronic sequence, Nb: number.

To understand the evolution of *fut8* genomic organization, we analyzed the repartition of intronic insertion sites in 56 complete orthologous *fut8* genes from organisms belonging to different animal phyla. As intron position analysis relies on consistent aa alignments, FUT8 sequences were aligned using Clustal Omega. The high aa identity observed in FUT8 catalytic domain (from exon 4 {E*4*l} to exon 11 {E*11*l}) allowed us to compare the intron-exon structure in this region. In contrast, the N-terminal region showed a very low level of aa conservation (**Figure S1**). As previously reported [44], when the insertion site was conserved, the intron phase was conserved as well. Several intron insertion sites were very well conserved (**Table 4** **and** **Table 6**), for instance i*3*l, i*5*l, i*7*l and i*9*l in chordata, hemichordata (*S. kowalevskii*) and echinodermata (*Strongylocentrotus purpuratus*). In nematoda, *fut8* gene was interrupted by 9 or 7 intronic sequences, but only one in rhabditida (*Caenorhabditis brenneri*) and three in trichocephalida (*Trichinella spiralis*) (*i.e.* i*9*l and i*6*l, i*7*l and i*9*l, respectively) were shared with *S. frugiperda fut8*. The intron sites i*7*l and i*9*l were also conserved in placozoa (*Trichoplax adhaerens*), cnidaria (*Hydra magnipapillata*) and annelida (*Capitella teleta*) (**Table 6**).

**Table 6:** Intron positions shared by *S. frugiperda fut8* (i*number*l) and with *fut8* genes identified in chordata (i*number*c).

Protein domain				Stem			Cys-rich				Motif I	Motif II		Motif III				
Intron insertion sites in *Sf*9 *fut8* gene				i*1*l	i*2*l	i*3*l	I*4*l		i*5*l	i*6*l	i*7*l	I*8*l		i*9*l	i*10*l	i*11*l	Total IS	Nb introns
Specific introns insertion sites in Chordata *fut8* gene								i*4*c					i*7*c				(bp)	
**Chordata**	Mammalia	Primate	*H. sapiens*			**X**		**+**	**X**		**X**		**+**	**X**			130,359	8
	Aves	Galliformes	*G. gallus*			**X**		**+**	**X**		**X**		**+**	**X**			36,774	8
			*M. gallopavo*			**X**		**+**	**X**		**X**		**+**	**X**			37,003	8
		Passeriformes	*T. guttata*			**X**		**+**	**X**		**X**		**+**	**X**			43,603	8
	Reptilia	Squamata	*A. carolinensis*			**X**		**+**	**X**		**X**		**+**	**X**			67,279	8
	Amphibia	Anura	*X. tropicalis*			**X**		**+**	**X**		**X**		**+**	**X**			72,102	8
	Actinopterygii	Cypriniformes	*D. rerio B*			**X**		**+**	**X**		**X**		**+**	**X**			88,524	8
		Tetraodontiformes	*T. rubipres*			**X**		**+**	**X**		**X**		**+**	**X**			23,935	8
		Beloniformes	*O. latipes*			**X**		**+**	**X**		**X**		**+**	**X**			44,674	8
		Perciformes	*O. niloticus*			**X**		**+**	**X**		**X**		**+**	**X**			45,900	8
**Echinodermata**	Echinoidea	Echinoida	*S. purpuratus*			**X**		**+**	**X**		**X**		**+**	**X**			58,902	8
**Hemichordata**	Enteropneusta	Enteropneusta	*S. kowalevskii A*			**X**		**+**	**X**		**X**		**+**	**X**			21,376	8
**Nematoda**	Secernantea	Trichocephalida	*T spiralis*							**X**	**X**			**X**			1,122	7
		Rhabditida	*C. brenneri*										**+**	**X**			652	9
		Spirudida	*L. loa*										**+**				3,347	9
			*B. malayi*										**+**				2,417	9
			*W. bancrofti*										**+**				2,954	10
		Ascaridida	*A suum*										**+**				13,589	10
**Annelida**	Polychaeta		*C. teleta*			**X**		**+**			**X**		**+**	**X**			1,183	7
**Placozoa**	Tricoplacia	Tricoplaciformes	T. adhaerens*								**X**		**+**	**X**			≥682	≥3
**Cnidaria**	Hydroida	Anthomedusae	*H. magnipapillata*								**X**		**+**	**X**			13,440	4
	Anthozoa	Actiniaria	*N. vectensis*								**X**		**+**	**X**			2,492	4

IS: Intronic Sequence.

(X): Presence of lepidopteran intron insertion site.

(**+**): Presence of chordata intron insertion site.

(*) The gene is truncated and the 5′ exon is missing.

In the *A. pisum* (arthropoda) and chordata *fut8* genes, exon *1-*58-*2* and exon *1-*50-*0* were flanked by the conserved intron sites i*7*l and i*9*l (**Figure 5**). Comparison of these exons with the corresponding lepidopteran exon 8 (*1-*51-*0*) and exon 9 (*0-*58-*0*) led us to hypothesize that these two exons derived from a common ancestral exon (*1-*109-*0*) that was found in *T. castaneum*. Similarly, the exon pairs *1-*55-*0*/*0-*62-*1* (E*4*l and E*5*l) in lepidoptera and *1-*38-*0*/*0-*78-*1* in chordata (E*4*c and E*5*c), which were flanked by the conserved intron sites i*3*l and i*5*l, could derive from a common ancestral exon.

## Discussion

FUT8 can be considered part of the GT-23 family within the CAZy classification, which groups enzymes acting on carbohydrates. This classification is based on the structural (presence of conserved peptide motifs) and mechanistic features (α1,6-fucosyltransferase) of these glycosyltransferases. GT-23 is a single-copy nuclear gene family, whereas most GT families are polygenic. *fut8* cDNAs have been cloned essentially from a few mammalian species (bovine, rat, pig, murine, and human, [15], [34], [35], [37], [45] and from two invertebrates, *Caenorhabditis elegans* and *Drosophila* [38]. In this work, we report the identification and molecular cloning of a *fut8* ortholog in the lepidoptera *S. frugiperda* and the analysis of the molecular function and genomic organization of this new lepidopteran gene.

All conserved motifs (I, II and III) and all essential aa residues implicated in FUT8 enzymatic activity were identified in the *Sf*9 FUT8 sequence. However, analysis of the cysteine residues involved in the disulfide bridges formation in most FUT8 proteins shows that one cysteine residue, which corresponds to Cys-472 in human FUT8, is missing in the lepidopteran protein. This change is accompanied by the presence of a new cysteine residue (Cys-438 in *S. frugiperda* FUT8) that is also conserved in many FUT8 sequences (tricocephalida, spirudida and ascaridia, *Ciona intestinalis*, *Nematostella vectensis* and in the hymenoptera FUT8 sequences analyzed in this study) (**Figure 1** **and Figure S1**). The high enzymatic activity measured with recombinant *Sf*9 FUT8 suggests that the disulfide bridge detected between Cys-465 and Cys-472 in most species can probably be equally established between Cys-465 with Cys-438 in *S. frugiperda* FUT8 without affecting its enzymatic activity or protein stability. Two potential *N*-glycosylation sites (Asn-167 and Asn-325) (**Figure 1**) are also present in the *Sf9* sequence, but these sites are not conserved in agreement with the fact that the enzyme activity is not dependent on the presence of *N*-glycans [15].

In an attempt to trace back the origin and evolutionary relationships of *fut8* genes, we identified *fut8* orthologs in a large panel of metazoan genomes and determined their gene organization.

Most GT families have significantly expanded in early vertebrates through whole genome duplications, and differential loss or retention of duplicated genes has contributed to the functional divergence of these GT families (e.g., sialyltransferases families) [46], [47]. GT-23 represents a unique case of an evolutionary ancient GT family that did not diverge during metazoan evolution. We have chosen to study its gene structure as a way to characterize the evolutionary relationships of these genes.

Comparative analysis of the *fut8* gene in various animal genomes provided few insights into the evolution of this single-copy gene in metazoans. In contrast with genes encoding α1,3-, α1,3/4- and α1,2-FucTs, all the characterized *fut8* genes presented a common poly-exon organization. Metazoan *fut8* genes contained a highly variable number of exons (from 12 exons in lepidoptera to 2 or 3 in diptera and no introns in chilopoda *Lepeophtheirus salmonis*), suggesting a complex evolutionary history with many intron gain and loss events. Analysis of the structure and evolution of the *fut8* genes in arthropods showed that the high evolutionary rates found for instance in the coleoptera and diptera branches (see branch length differences between *Drosophila* and *Aedes aegypti* in **Figure 4**) corresponded to differences in gene organization as well.

The total size of the intronic sequences inserted in *fut8* genes varied greatly among insects with more than 10,000 bp in lepidoptera and only 160 bp in diptera (culicidae), with the notable exception of *A. aegypti*, which presented 13,978 bp of intronic sequences. This observation is in agreement with the analysis of the average intron size of the *A. aegypti* genome [48] that shows a 4-fold increase due to intron infiltration by transposable elements compared to other diptera species, such as *A. gambiae* and *D. melanogaster*. Similarly, the intronic sequences identified in *D. plexippus fut8* were about two times smaller than in *B. mori* and *S. frugiperda*, possibly because *D. plexippus* has the smallest genome among lepidoptera [49]. The same diversity was observed in other phyla. For instance, in nematoda, *fut8* has either 7 or 10 introns with relatively short intronic sequences (from 652 bp in *C. brenneri* to 3,676 bp for *Caenorhabditis japonica*). In placozoa and cnidaria, only 3 or 4 introns were found for a total of 682 bp of intronic sequences in *T. adhaerens fut8* and 13,440 bp in *H. magnipapillata fut8*. In vertebrates, the intron number appeared to be constant (8 introns). The main difference, compared to other phyla, was the very large total size of intronic sequences (about 130,000 bp in *Homo sapiens* and *Rattus norvegicus* 173,134 bp in *Bos taurus)* (**Table 4** **and** **Table 6**).

Intron insertion sites in phase 0 were over-represented in gene sequences encoding the most conserved part of the FUT8 proteins: i*4*l and i*4*c in the cysteine-rich domain, and i*8*l (motif II), i*9*l (motif III), i*10*l (SH3) and i*11*l (SH3) in the catalytic domain. Moreover, in human FUT8 structure [9], introns i*4*c and i*8*c/i*9*l are located at the C- and N- terminal domain of alpha helix 3 and alpha helix 11 respectively. Such features are proposed to be characteristic of ancient conserved genes [50].

Many exons were shared by different arthropoda orders, such as phtiraptera, coleoptera, hemiptera, diptera, lepidoptera and even crustacea (**Figure 5**). Remarkably, despite the high conservation of lepidoptera FUT8 sequence with vertebrates and hymenoptera (*S. frugiperda* FUT8 sequence shares 44.24% and 45.93% aa identity with *H. sapiens* and *A. mellifera* respectively), the exon-intron organization of hymenoptera *fut8* genes is order-specific with no shared exons. This is particularly surprising because when the analysis is extended to orthologous *fut8* genes of other phyla, many *Spodoptera* intron insertion sites are still conserved throughout the animal kingdom. Particularly, intron positions i*7*l and i*9*l are common to most of the *fut8* genes analyzed in this work (**Table 4** **and** **Table 6**). As these introns are located in a gene portion encoding motif I and III respectively (highly conserved regions of FUT8), a high selection pressure could be exerted on these parts, such as for instance the presence of regulatory elements that are important for gene expression. The i*8*l insertion site is only found in lepidoptera and some diptera, such as culicidae and psychodoidea (*Lutzomyia longipalpis*). This site is very close to the chordate site i*7*c with only 24 nucleotides between these two sites (**Figure S1**) forming a “near intron pair” as defined by [51]. In contrast to i*8*l, i*7*c is conserved in many arthropods (*A. pisum, P. humanus, D. pulex, Strigamia maritima*), cnidarians (*N. vectensis and H. magnipapillata*) and nematodes (*Brugia malayi and C. brenneri*). Thus, position i*7*c appeared before i*8*l and we hypothesize that the i*8*l site might have resulted from i*7*c sliding. Similarly, the chordate insertion site i*4*c is found in the arthropod *S. maritima* (**Figure S1**). Comparison of the lepidoptera and chordata exon pairs *1-*55-*0*/*0-*62-*1* and *1-*38-*0*/*0-*78-*1,* located between the conserved sites i*3*l and i*5*l, suggests the existence of a common ancestral exon. As hypothesized for i*8*l, position i*4*l could have derived from the sliding of the ancestral position i*4*c (51 nt between i*4*l and i*4*c). However, unlike i*7*c, i*4*c is not well conserved outside chordates (**Figure 5**).

**Figure 6:**
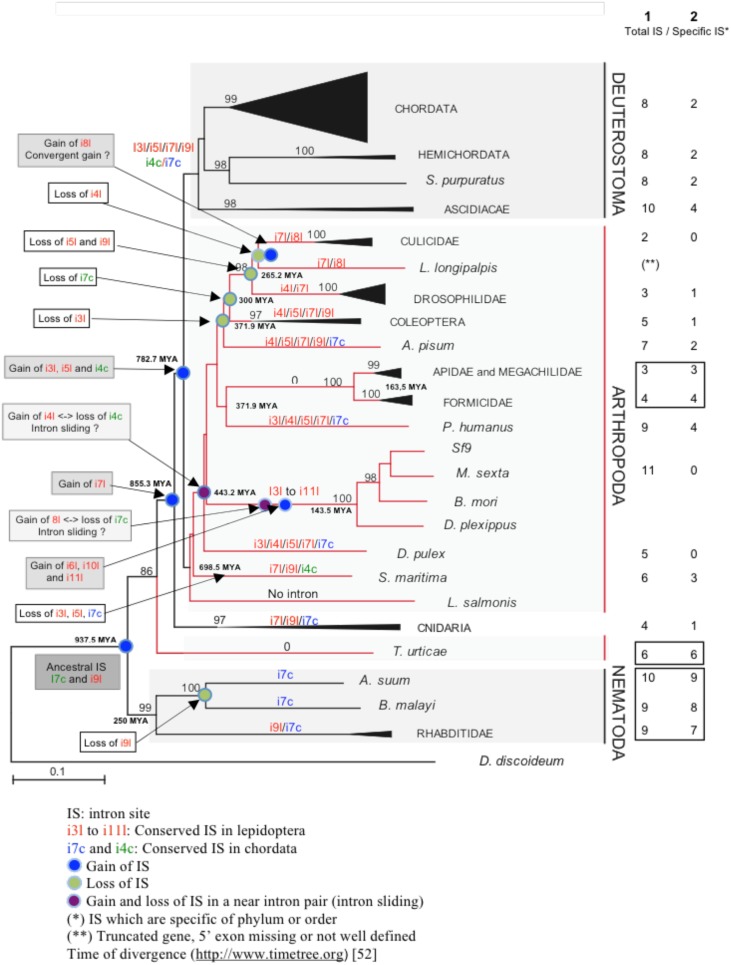
Schematic illustration of the correlation between animal *fut8* gene phylogeny, intron gain/loss and intron position (gene organization). This analysis was carried out by considering only the intron insertion sites in the *fut8* gene sequences encoding the conserved region of FUT8 proteins, between i*3*l and the stop codon. On the right, column 1 shows the total number of intron insertion sites (IS) identified in the different *fut8* genes, and column 2 shows the number of order- or family-specific intron insertion sites. Intron gains and losses are highlighted (grey and white boxes, respectively) as well as putative intron sliding in “near-intron-pairs” (light grey boxes). Putative ancestral introns are in a dark grey box.

To conclude, we propose a model of *fut8* gene evolution in animals (**Figure 6**). In this model, i*7*c and i*9*l are considered as ancestral intron insertion sites. Until the arthropoda-chordata split, intron gain seems to have been the most favored event with gain of i*7*l about 855MYA (Million years ago) and of i*3*l, i*5*l, i*4*c about 783 MYA [52]. After this divergence, intron loss seems to have become rather more common with the consecutive loss in arthropods of i*3*l (372 MYA), i*7*c (300 MYA), i*5*l and i*9*l (265 MYA). These intron losses could be accompanied by specific intron site gains (for instance, gain of i*6*l, i*10*l and i*11*l in lepidoptera) (**Figure 6**, compare lane 1 and 2) or not (for instance in *D. pulex)*. It has to be noted that the intron site gains and losses identified in arthropods are not observed in chordates. Indeed, insertions of spliceosomal introns are rarely observed during evolution of vertebrates [53]. As *fut8* intron-exon organization is order-specific, these intron losses and gains may be linked to evolutionary innovations, such as appearance of new orders.

Finally, the gain of some intron sites, such as i*8*l in some diptera (culicidae, *C. pipiens* and psychodidae, *L. longipalpis*), i*10*l in *C. intestinalis* and i*6*l in *T. spiralis* (**Figure 6** **, Figure S1,** **Table 4** **and** **Table 6**), may be explained by convergent/parallel intron gains, as recently described by [54] for hymenopteran paralogs, suggesting the presence of intron insertion hot spots [55].

Unlike other well studied glycosyltransferases [46], [47], we found that this single-copy gene characterized by highly conserved motifs is present from the very first metazoans to vertebrates. This could be explained by FUT8 important function. Evolution of *fut8* gene organization is also very specific and may accommodate different way of regulating its expression for instance in response to the environment of these insects.

## Materials and Methods

### Cells and viruses

The *Sf*9 subclone of the *S. frugiperda Sf*21 cell line [56] was maintained at 28°C in TC100 medium (GIBCO) containing 5% heat-inactivated fetal calf serum. Cells were infected with baculoviruses *Ac*MNPV clone 1.2 [57] at a multiplicity of infection (MOI) of 2 plaque-forming units (PFU) per cell. After 1 h incubation at room temperature with the viral suspension, fresh culture medium was added and cells were incubated at 28°C for 5 days. The viral titers were determined by plaque assay [58].

### DNA and RNA purification

High molecular weight genomic DNA was extracted from *Sf*9 cells (15×10^6^ cells) using the Genomic-Tip Kit (QIAGEN) as recommended by the manufacturer. Total cytoplasmic RNA was extracted from 2×10^7^ *Sf*9 cells using a method previously described [59]. After precipitation, RNA pellets were washed with a cold solution containing 75% ethanol and 25% sodium acetate and resuspended in 50 µl of distilled water. Fifty µg of RNA were then incubated with 15 units of RNase-free DNase I (New England Biolabs) at 37°C for 20 min and then the DNase was inactivated with 5 mM EDTA at 75°C for 10 min. RNA samples were loaded on an RNeasy column (QIAGEN) as recommended by the manufacturer. Purified RNA was immediately stored at −80°C.

### Cloning of the *Sf9* α1,6-FucT cDNA

*Sf9 fut8* cDNA was cloned by RT-PCR using degenerate primers [60]. The two *ForFut1deg* and *BacFut2deg* degenerate primers were designed based on the conserved aa sequence found in five species in which *fut8* cDNA had been already isolated and characterized: *H. sapiens* (AJ536056), *B. taurus* (BC104555), *D*. *melanogaster* (AF441264), *Drosophila yakuba* (AJ830719) and *Drosophila pseudoobscura* (AJ830720) (**Table S1**, for primer sequences) (Eurogentech, Belgium). RT was performed as follows: a mixture (final volume of 20 µl) including 1 µg of total RNA from *Sf9* cells, 4 µl of 10 X RT buffer (QIAGEN), 4 µl of 5 mM of each dNTP (QIAGEN), 4 µl of 10 pmoles/ml *BacFut2deg*, 20 units of RNase inhibitor (Roche) and 8 units of Omniscript reverse transcriptase (QIAGEN) was incubated at 37°C for 1 hour. The reverse transcriptase was inactivated at 93°C for 5 min.

Polymerase chain reaction (PCR) amplification was then carried out in a final volume of 20 µl containing 2 µl of 10 X Vent DNA polymerase (New England Biolabs), 2 µl of 10 mM each dNTP (Biolabs), 20 pmoles of each degenerate primer, 1.5 µl of 25 mM MgSO_4_, 1 unit of Vent Polymerase and 0.5 µl of the RT mixture. Thirty-five cycles of amplification were performed on a Mastercycler apparatus (Eppendorf): denaturation at 94°C for 30 sec, annealing for 1 min at a temperature depending on the primer used, extension at 72°C for 1 min and a final extension of 10 min at 72°C.

PCR products were separated on low melting agarose gels (FMC, Nusieve GTG) and purified with the QIAquick Gel Extraction Kit (QIAGEN). The 5′-ends were adenylated by incubating the DNA with 1 unit of Taq DNA Polymerase (Biolabs) and 5 µl of 10X ThermoPol buffer (20 mM Tris-HCl pH 8.8, 10 mM KCl, 10 mM (NH_4_)_2_SO_4_, 2 mM MgSO_4_, 0.1% Triton X-100) and 200 µM dATP (final volume: 50 µl) at 72°C for 20 min. The PCR fragments were purified with the QIAquick PCR Purification Kit (QIAGEN) before ligation in the pGEM-T Easy vector system (Promega) for sequencing.

The 5′- and 3′- ends of the cDNA were amplified using the 5′/3′-RACE Kit, 2^nd^ generation (Roche), following the manufacturer’s protocol, and the exact-match primers *Bac5′RACEFut8* and *For3′RACEFut8* (**Table S1**), designed based on the *fut8* cDNA sequence obtained with the degenerate primers. The AMV reverse transcriptase and total RNA from *Sf*9 cells, as before, were used for the RT step. Briefly, 3′-RACE uses the polyA stretch generally present at the 3′-end of each mRNA as hybridization site for the oligo(dT) anchor primer. To specifically amplify the 3′ *fut8* cDNA end, RNA was thus reverse transcribed with the oligo(dT) anchor primer and then the RT product was PCR amplified with *For3′RACEFut8* and the oligo(dT) primers. For the 5′-RACE, the specific *Bac5′RACEFut8* primer was used in the RT step. Then, to specifically amplify the 5′-end of the *fut8* cDNA, a polyA stretch was added at the 5′-end of the cDNA by a terminal transferase and used as a hybridization site for the oligo(dT) anchor primer. The entire 1734 bp ORF was reconstituted in the pGEM-T Easy plasmid, giving the final pGEMFUT8*Sf* construct.

### Genomic DNA analysis

*Sf*9 cell genomic DNA (10** µ**g) was digested with *Eco*RI or *Hind*III restriction endonucleases. DNA fragments were then separated by electrophoresis on 0.9% agarose gels (SeaKem GTG, Lonza) and transferred to positively charged nylon membranes (Roche). Membranes were pre-hybridized at 68°C in 5X SSC, 0.1% N-laurylsarcosine, 0.02% (w/v) SDS, 1% blocking reagent (Roche) and 100** µ**g**/**ml calf thymus DNA for 3 hours. Overnight hybridization was performed at 68°C in the same buffer with a 204****bp *fut8* specific probe (nucleotide 217–420 of the *fut8* cDNA cloned in pGEM-T Easy, **Figure 1**) labeled with digoxigenin using the PCR DIG Probe Synthesis Kit (Roche). After hybridization, filters were washed twice at room temperature with 2X SSC, 0.1% SDS for 5****min and then twice with 0.1X SSC, 01% SDS at 68°C for 15****minutes. Probe-target hybrids were revealed by incubating the membranes with an alkaline-phosphatase conjugated anti-digoxigenin antibody (Roche) and by a chemoluminescent reaction using CSPD as substrate (Roche). Membranes were exposed to Kodak films for 20****min.

### RNA analysis

Total RNA from *Sf*9 cells was resolved (1 µg per lane) on 1% agarose gels and transferred to positively charged nylon membranes (Roche). A digoxigenin-labeled riboprobe was synthesized using the DIG Northern Starter Kit (Roche) using as a template the complete FUT8 coding sequence (pos. -1 to pos. 1686 in the *fut8* cDNA, **Figure 1**) cloned in pGEM-T Easy (pGEMFUT8*Sf)*. Hybridization and revelation were performed as described for Southern blotting.

### PCR analysis of the genomic structure of the *Sf9* α1,6-FucT gene

PCR amplifications were performed using *Sf9* genomic DNA as a template. DNA was isolated as described above using the Genomic-Tip Kit (QIAGEN) and resuspended in water. PCR amplifications were carried out in a final volume of 50 µl containing 10 µl of 5X Phusion DNA polymerase buffer (HF buffer) (Finnzymes, Finland), 1 µl of 10 mM each dNTP (Biolabs), 0.5 µmoles of each primer (**Table S1**; Cloning of introns), 1 unit of Phusion DNA Polymerase and 200 ng of *Sf9* genomic DNA. After denaturation at 98°C for 2 min, a two-step protocol was used with (i) seven cycles of amplification in the following conditions: denaturation at 98°C for 15 sec, annealing and extension for 3 minutes at 72°C and (ii) thirty cycles of amplification with denaturation at 98°C for 15 sec, annealing and extension for 5 min at 68°C. PCR products were isolated on agarose gels (SeaKem GTG, Lonza) and purified with the QIAquick Gel Extraction Kit (QIAGEN). PCR fragments were then adenylated with Taq Polymerase as described above and cloned in pGEM-T Easy (Promega) for sequencing analysis (Operon Eurofins MWG, Germany).

### Construction of the recombinant baculovirus expressing soluble and tagged *Sf9* FUT8

To produce soluble recombinant FUT8, the 5′-end of the cDNA encoding the N-terminal domain of FUT8 (from aa 1 to aa 30 at the end of the putative transmembrane domain) was deleted and replaced by the signal peptide sequence of the baculovirus ecdysteroid glycosyltransferase (EGT) [61] followed by a sequence encoding the FLAG epitope (Asp-Tyr-Lys-Asp-Asp-Asp-Asp-Lys) to produce a recombinant protein that can be easily purified and that can be differentiated from the endogenous protein. To this aim, a *Hpa*I-*Bgl*II DNA fragment encoding the FLAG epitope and aa 31 to aa 40 of *Sf9* FUT8 was synthesized using overlapping oligonucleotides and inserted in frame with the EGT signal peptide present in the pUC PS-EGT vector (pUC PS-EGT-FLAG-NterFut8*Sf* construct) [62]. Then, the C-terminal domain of *fut8* cDNA was isolated from the pGEMFut8*Sf* plasmid by *EcoR*I-*BamH*I digestion and inserted in the pUC PS-EGT-FLAG-NterFut8*Sf* construct to obtain the pUC PS-EGT-FLAG-Fut8*Sf* construct. After *BamH*I and *Hind*III digestion, the 1722 bp fragment containing the EGT-FLAG-Fut8 cDNA was isolated by agarose gel electrophoresis (Nusieve GTG, Lonza), purified using the QIAGEN Gel Extraction Kit and ligated in the p119 baculovirus transfer vector digested with *Bgl*II and *Hind*III to produce the p119-EGT-FLAG-Fut8*Sf* construct.

*Sf9* cells (4×10^6^ cells) were seeded in 25 cm^2^ flasks and co-transfected with 500 ng of purified *Ac*SLP10 viral DNA [59] and 5 µg of p119-EGT-FLAG-Fut8*Sf* by lipofection [63] with DOTAP (Roche). The p119 transfer vector is designed for recombination in the P10 locus. *Ac*SLP10 is derived from wild type *Ac*MNPV 1.2 [57] and has only one strong promoter (P10) to drive the expression of a polyhedrin gene [59]. After 5 days of incubation at 28°C, recombinant (i.e., polyhedron-negative) viruses were purified by plaque assay [58]. Viral stocks were generated by propagating viruses in *Sf9* cells (75 cm^2^ flasks) and titrated using plaque assays.

### Production and purification of soluble FUT8

*Sf9* cells seeded in roller bottles at a density of 400,000 cells/mL in serum-free medium were infected at a MOI of 2 PFU per cell. After 3-day incubation at 28°C, supernatants were collected and stored at −80°C before use. Supernatants were then concentrated and diafiltered against TS buffer (50 mM Tris-HCl pH 7.4, 150 mM NaCl) using Centromate cassettes (Pall, 0.1 m^2^/30 kDa) before loading on a 5 ml column of anti-FLAG M2 Affinity gel (Sigma) equilibrated with TS buffer. The FLAG-tagged protein was eluted with 30 ml of 100 µg/ml FLAG peptide (in TS buffer). Fractions containing the purified protein were concentrated (AMICON Ultra 50 k, Millipore) and analyzed by PAGE and western blotting.

### FUT8 assays

After 2 hours of dialysis against 50 mM sodium acetate buffer (pH 7.5), 5 mg of human apotransferrin (Sigma) were incubated with 120 mU *Arthrobacter urefasciens* neuraminidase (Sigma) and 50 mU *Escherichia coli* β-galactosidase (Sigma) in a final volume of 10 ml at 37°C for 24 hours. After 4 hours dialysis against water, asialo-agalacto apotransferrin was freeze-dried. The absence of galactose and sialic acid residues was verified by gas chromatography-mass spectrometry (GC-MS). To test the enzymatic activity of recombinant *Sf9* FUT8, assays were performed in 50 µl total volume that included 20 µl of purified recombinant protein solution (140 µg/ml), 70 mM cacodylate buffer (pH 7.2) 10 mM L-fucose, 6 mM GDP-[^14^C]-L-fucose (299 mCi/mMol, Amersham), 10 mM GDP-fucose and 100 µg asialo-agalacto apotransferrin as acceptor, as previously described [34]. After incubation at 30°C for 4 hours, the reaction was stopped with 150 µl of water and samples were precipitated with 1 ml of 5% PTA and processed for scintillation counting. The transfer of [^14^C]-fucose is expressed in cpm.

### *In silico* α1,6-FucT sequences retrieval and phylogenetic analysis

Only eukaryotic core α1,6-FucT sequences were considered for this study. Homologous *fut8* sequences were searched by querying all genomic and expressed sequence tag (EST) divisions of the National Center for Biotechnology Information (NCBI database, Washington, DC, USA), as described previously for sialyltransferases [22], [64] (**Table S2**). DNA and aa sequences were analyzed using DNA Strider [65]. PSORT II (http://expasy.org) was used for the prediction of protein localization sites in cells. Homology searches were performed using the BLAST program [39]. Multiple sequence alignments of the 86 deduced aa sequences homologous to α1,6-fucosyltranferases were generated using ClustalW 2.0.8, Clustal Omega 1.1.0 or MAFFT, which are multiple sequence alignment programs available at http://www.ebi.ac.uk. Gblocks (http://molevol.cmima.csic.es/castresana/Gblocks_server.html) [66] was used to select the 336/785 informative sites. Evolutionary analyses were conducted using the Neighbor Joining (NJ) and Maximum Likelihood (ML) methods implemented in MEGA 5.05 [67]. The branch robustness was tested with 1050 bootstrap replicates. Conservation of synteny and gene order in the metazoan were visualized at the Genomicus web site (version 19.01) (http://www.genomicus.biologie.ens.fr/genomicus-metazoa-19.01/cgi-bin/search.pl) [42].

## Supporting Information

Figure S1 **Amino acid sequences of α1,6-fucosyltranferases from different phyla were aligned using Clustal Omega 1.1.0 (** www.ebi.ac.uk). Letters on grey background indicate the position of intron insertion in the genes. Numbers indicate the intron phase. When the insertion phase is 1 or 2, the aa corresponding to the split codon is in highlighted in grey, when the insertion phase is 0, the two flanking aa are in grey. Putative transmembrane domains determined using http://wolfpsort.org are underlined. Conserved cysteine residues are highlighted in yellow.
(PDF)

Figure S2 **The evolutionary history was inferred using the Neighbor-Joining method [68] and 86 amino acid sequences aligned with MAFFT (EBI).** All positions containing gaps and missing data were eliminated. The final dataset contained 461 positions selected in 17 blocks by GBlocks [66] (58% of the original 785 positions). The *Dictyostelium discoideum* sequence was used as outgroup. The optimal tree with a branch length sum = 6.93435487 is shown. The percentage of replicated trees (>75%) in which the associated taxa clustered together in the bootstrap test (1050 replicates) is shown next to the branches. The tree is drawn to scale, with branch lengths (next to the branches) in the same units as those of the evolutionary distances used to infer the phylogenetic tree. The evolutionary distances were computed using the p-distance method [69] and refers to the number of amino acid differences per site.
(PDF)

Table S1 **Primers used in this study.**
(PDF)

Table S2 **Nomenclature, name and GenBank accession number of the** ***fut8*** **gene sequences used in this study.** (*) Nucleotide sequence data available in the Third Party Annotation Section of the DDBJ/EMBL/GenBank databases. (**) Ensembl accession number.
(PDF)
