# Correction: The α1,6-Fucosyltransferase Gene (*fut8*) from the *Sf9* Lepidopteran Insect Cell Line: Insights into *fut8* Evolution

**DOI:** 10.1371/journal.pone.0122944

**Published:** 2015-04-07

**Authors:** The PLOS ONE Staff

There is an error in the legend for Fig. 3, "Conserved aa and motifs found in all the α1,6-fucosyltransferases sequences". Please view Fig. 3 and its complete, correct legend here:

**Fig 3:**
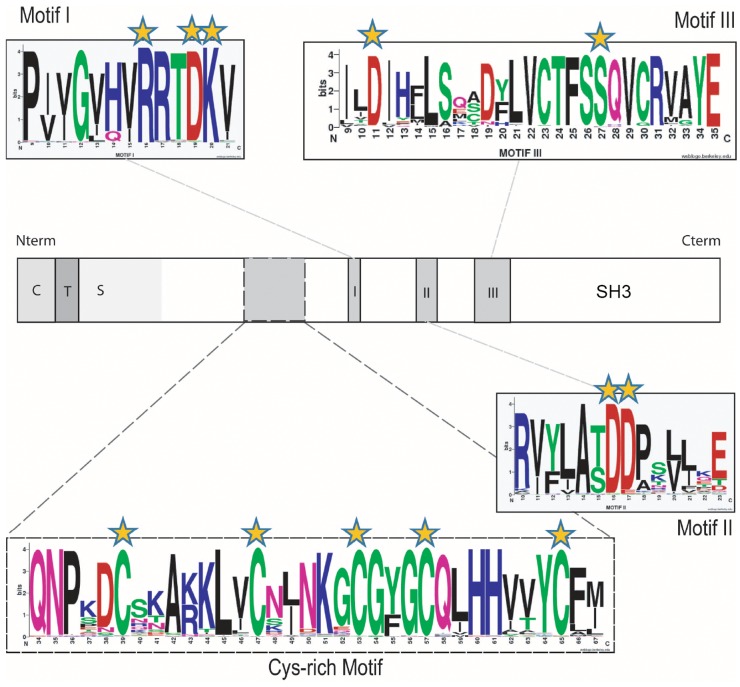
Conserved aa and motifs found in all the α1,6-fucosyltransferases sequences. Schematic representations of the FUT8 protein showing the cytoplasmic (C), transmembrane (T) and stem region (S) characteristic of α1,6-fucosyltransferases. The catalytic domain is in white and motifs I, II and III in grey. In addition, a region found only in α1,6-fucosyltransferase with conserved cysteine residues is indicated by dashed lines and was named "Cys-rich" domain. Conserved aa and those implicated in the enzymatic activity are highlighted with orange stars. The conserved peptide sequences used to generate the motif I, motif II and motif III sequence logos were extracted from multiple alignments of 96 α1,6-fucosyltransferase sequences identified in the databases (Table S2) and visualized at the Weblogos site at Berkeley, as described previously [63]. In the logos, aa are colored according to their chemical properties: polar aa (G, C, S, T, Y) are green, basic (K, R, H) are blue, acidic (D, E) are red, hydrophobic (A, V, L, I, P, W, F, M) are black and neutral polar aa (N, Q) are pink. The overall height of the stacks indicates the sequence conservation at a given position, while the height of the symbol within the stack indicates the relative frequency of each aa at that position. [70, 71]
