# Supplementary material for: The α1,6-Fucosyltransferase Gene (fut8) from the Sf9 Lepidopteran Insect Cell Line: Insights into fut8 Evolution
Source: PLoS One. 2014 Oct 21;9(10):e110422. doi: 10.1371/journal.pone.0110422 (PMC4204859; doi:10.1371/journal.pone.0110422)
Supplement: Figure S1 — Amino acid sequences of α1,6-fucosyltranferases from different phyla were aligned using Clustal Omega 1.1.0 ( www.ebi.ac.uk). Letters on grey background indicate the position of intron insertion in the genes. Numbers indicate the intron phase. When the insertion phase is 1 or 2, the aa corresponding to the split codon is in highlighted in grey, when the insertion phase is 0, the two flanking aa are in grey. Putative transmembrane domains determined using http://wolfpsort.org are underlined. Conserved cysteine residues are highlighted in yellow. (PDF) [file pone.0110422.s001.pdf]

Figure S1

**Nematoda**	Fut8Cjaponica	------------MSVFHQFPPNISHFRSPASRSDMIKCIAVVGTVI----------WMTM
	Fut8Celegans	----------------------------------MLKCIAAVGTVV----------WMTM
	Fut8Cbrenneri	----------------------------------MFKCIAAVGTVV----------WMTM
	Fut8Cremanei	----------------------------------MFKCIAAVGTVV----------WMTM
	Fut8Asuum	----------------------------MQQLGCSMRRAAMAAVAV----------WVLI
	Fut8Lloa	-----------------------MKLR-RTSCSCSMHCILITAVVI----------WSSI
	Fut8Bmalayi	-----------------------MKLR-RTVCPCSMHCILISAVVI----------WSSI
	Fut8Wbancrofti	-----------------------MKLR-RTGCSCSMHCILITAVVI----------WSSI
	Fut8Tspiralis	--
**Arthropoda/trombidiformes**	Fut8Turticae	----------------------------MALNFAYAGRVLVGVVLV----------WLLL
**Cnidaria**	Fut8Hmagnipapillata	-----------------------------MAAKIM--RYIGFVTVL----------WLCV
	Fut8Nvectensis	-----------------------------MTRRSW--RYLLILLTI----------WLLI
**Arthropoda/Mesostigmata**	Fut8Moccidentalis	-----------------------MARSIPLTDSTWMSNAIHDYAAASDPPGYHIVSGVAL
**Chordata**	Fut8CintestinalisA	--------------------------ML---RRGQRVFIFIGILLS----------ALNI
**Arthropoda/Maxillopoda**	Fut8Lsalmonis	---------------------------MR--CKSIHFFFVFIILWI----------WLMI
**Echinoderma**	Fut8SpurpuratusA	--
**Annelida**	Fut8Cteleta	------------------------------MRGTW--RVVAGLLVF----------WLLV
Hemichordata	Fut8SkowalevskiiA	------------------------------MKTLW--KFVIALLFF----------WFVI
**Cephalochordata**	Fut8Bfloridae	--
**Chordata**	Fut8Xtropicalis	---------------------------MRPWTGSW--RWIMLILFA----------WGTL
	Fut8Rnorvegicus	---------------------------MRAWTGSW--RWIMLILFA----------WGTL
	Fut8Hsapiens	---------------------------MRPWTGSW--RWIMLILFA----------WGTL
	Fut8Btaurus	---------------------------MRPWTGSW--RWIMLILFA----------WGTL
	Fut8Acarolinensis	---------------------------MRPWTGSW--RWIMLILFA----------WGTL
	Fut8Ggallus	---------------------------MRPWTGSW--RWIMLILFA----------WGTL
	Fut8Mgallopavo	---------------------------MRPWTGSW--RWIMLILFA----------WGTL
	Fut8Tguttata	---------------------------MRPWTGSW--RWIMLILFA----------WGTL
	Fut8DrerioB	---------------------------MRPWTGSW--RWIALVLLA----------WGTL
	Fut8Trubipres	---------------------------MRPWAGSW--RWITLVLLA----------WGTL
	Fut8Oniloticus	---------------------------MRPWAGSW--RWITLVLLA----------WGTL
	Fut8Olatipes	---------------------------MRPWAGSW--RWITLVLLA----------WGTL
**Arthropodes/Lepidoptera**	Fut8Hmelpomene	-----------------------------MYLSKWK-RVTVVLLLV----------WIIV
	Fut8Dplexippus	-----------------------------MYLTKWK-RAAVLLLFI----------WIAV
	Fut8Bmori	-----------------------------MYFAKWK-RVAVVLLAV----------WIVV
	Fut8Sfrugiperda	-----------------------------MYLAKWK-RAAVVLLFV----------WIVV
	Fut8Msexta	-----------------------------MYLAKWK-RVAVVLLVI----------WIVV
**Arthropodes/Hymenoptera**	Fut8Mrotundata	------------------------MATFWSGRPGWLGKVGIVLLAT----------WLLV
	Fut8Amellifera	------------------------MATFWSGRPGWLGKVGLALLAT----------WLLI
	Fut8Bimpatiens	------------------------MATFWSGRPGWLGKVGIALLAT----------WLLV
	Fut8Hsaltator	------------------------MAAICSGRLGWLGKIVIALLAT----------WLIA
	FUT8Cfloridanus	------------------------MLAICSGRPGWLGKLGIALLIT----------WFFV
	Fut8Sinvicta	------------------------MAAICSGRPGWLGKLGIALLAT----------WFLV
	Fut8Acephalotes	------------------------MAAICSGRPGWLGKLGIALLAA----------WFLV
	FUT8Aechinatior	------------------------MAAICSGRPGWLGKLGIALLAA----------WFLV
**Arthropodes/Phtiraptera**	Fut8Phumanus	0 MEHKKVYSKCINLNLINVIFSYEVLCNKAMRSQICS-RILLVSFIV----------WFII
**Arthropodes/Chilopoda**	Fut8Smaritima	------------------------------MAVGVG-KAIVVLLAV----------WLII
**Arthropodes/Brachiopoda**	Fut8Dpulex	-----------------------MTMNLRSSNKILA-QAAIILFFF----------WVVA
**Arthropodes/Hemiptera**	Fut8Apisum	-----------------------------MRQQGWL-RILAFLAFS----------WVAF
**Arthropodes/Coleoptera**	Fut8Tcastaneum	----------------------MDTILRRINVVGWH-RMLVVFLFI----------WLVI
**Arthropodes/Diptera**	Fut8Dmojavensis	------------ML--------LVRQLFGSSSNSWV-RALIIFVLA----------WAVL
	Fut8Dpseudo	------------ML--------LVRQLFGASANSWV-RALVIFVLA----------WVAL
	Fut8Dananassae	------------ML--------LVRQLFGASANSWA-RALIFFVLA----------WVAL
	Fut8Dmelanogaster	------------ML--------LVRQLFGASANSWA-RALIIFVLA----------WIGL
	Fut8Agambiae	-------------M--------IVRQLMG--LNPWA-RVLVPFLFV----------WVLF
	Fut8Aaegypti	-------------M--------ILRQLMG--LNTWV-RVLIGFISV----------WVIL
	Fut8Cpipiens	-------------M--------ILRQLMG--LNTWV-RVLIAFISV----------WVIL

```
Fut8Cjaponica          1 FLFLYLQLSNNN----------------TS-----GGDTI------RAWRQTKEAIERLQ
Fut8Celegans           1 FLFLYSQLSNNQ-----------------S-----GGDSI------RAWRQTKEAIDKLQ
Fut8Cbrenneri          1 FLFLYLQLSNNQ----------------TS-----GGDSI------RAWRQTKEAIDKLQ
Fut8Cremanei           1 FLFLYLQLSNNQ----------------SS-----GGDSI------RAWRQTKEAIDKLQ
Fut8Asuum              1 LIYLSIGIFTIQ----------------SR-----EAAERQNEDLFVKYGKAMKEVGVLR
Fut8Lloa               1 FFYLSLSLYGLQ----------------SK-----GKDVNEQQALILNYERALLDLAELK
Fut8Bmalayi            1 FFYLSFNLYGLQ----------------SK-----GKDINEERILALNYERALLDLAELK
Fut8Wbancrofti         1 FFYLSFNLYGLQ----------------SK-----GKDVNEQHALALNYERALLDLAELK
Fut8Tspiralis            ------------------MFCSAEVEFFS--AADPYYNSDVDQHLLKRLEEAIAEVNTLR
Fut8Turticae             IFFIGGPLLKRTEG------------------DYSLINSENGELILARLSRASSELDSLR
Fut8Hmagnipapillata      LLYMGFNVYQLV---------------------------EESRKSTHELSMVVEKVAHLK
Fut8Nvectensis           LAYMGWNLLVLV---------------------------EDSNKNQAALDRKDRHVDLLN
Fut8Moccidentalis      0 SCYPNERLVPAARNFGGSDHCELQHRVMSMRSRNRGKLYPDRFGIDSRVNRALDELEDLK
Fut8CintestinalisA     2 FITWKSCVTSSE----------------------------FSNAQQSQQTHVDVILEQLQ
Fut8Lsalmonis            KFMWSSKTSSQL-----------------------DNSPEFSEDLLKQLEEAQKQIERLT
Fut8SpurpuratusA         ------MVVED-----------------------------AHDNNQRNVENGIFDIENMG
Fut8Cteleta              LLYMSATLFQGG----------------S---------DSTADRTERQLSRALHELDLLK
Fut8SkowalevskiiA        LLYLGGAIGPN----------------------------TEIKLSSRDLNKMISEIELMK
Fut8Bfloridae            --MLGNFY--TG---------------------------DPSTKSSHDLARALAELEKMK
Fut8Xtropicalis          LFYIGGHLVRDN---------------------------ENPDHSSRELSKILAKLERLK
Fut8Rnorvegicus          LFYIGGHLVRDN---------------------------DHPDHSSRELSKILAKLERLK
Fut8Hsapiens             LFYIGGHLVRDN---------------------------DHPDHSSRELSKILAKLERLK
Fut8Btaurus              LFYIGGHLVRGN---------------------------DHPDHSSRELSKILAKLERLK
Fut8Acarolinensis        LFYIGGHLVRDN---------------------------EHPDHSSRELSKILAKLERLK
Fut8Ggallus              LFYIGGHLVRDS---------------------------EHPDHSSRELSKILAKLERLK
Fut8Mgallopavo           LFYIGGHLVRDS---------------------------EHPDHSSRELSKILAKLERLK
Fut8Tguttata             LFYIGGHLVRDS---------------------------EHPDHSSRELSKILAKLERLK
Fut8DrerioB              LFYIGGHLVKDS---------------------------EHAPRSSRELAKILTKLERLK
Fut8Trubipres            LFYIGGHLVRDS---------------------------EHPERSSRELSKILAKLERLK
Fut8Oniloticus           LFYIGGHLVRDS---------------------------EHPERSSRELSKILAKLERLK
Fut8Olatipes             LFYIGGHLVRDS---------------------------EHPERSSRELSKILAKLERLK
Fut8Hmelpomene           TYLVISPLRCEG----------------G---------TEEAADFQERLKTVSQQLESLK
Fut8Dplexippus           TYLVISPLRCDS----------------S---------SEESIDFQERLKTASLQLELLK
Fut8Bmori                TYLVISPLRCSG----------------N---------SDEIPDVQERLKRMSSELEVLW
Fut8Sfrugiperda          TYLVISPLRCNG----------------N---------QDEVAEFQERLKKVSSQLETLR
Fut8Msexta               TYLVISPLRCNG----------------S---------ADEAPEFQERLKRVSSQLETLR
Fut8Mrotundata           LIVSVSHIFKAN----------------SLSSNN---ESPTNKENAQRLAQMVNDFEILK
Fut8Amellifera           LIISVSHIFKTN----------------NLSSNN---ESPTNKENAQRLAQMVNDFEILK
Fut8Bimpatiens           LIISVSHIFKAN----------------NLSSNN---ESPTNKENAQRLAQMVNDFEILK
Fut8Hsaltator            LIISISHIFKSN----------------ISSNQD---ANAVNKVNMQRLAKMVNDFEILK
FUT8Cfloridanus          LILSIVHIFKLN----------------SLSSRD---ASTANKENTQRLAQMVNDFEILK
Fut8Sinvicta             LIITIVHIFKSN----------------SLSSRD---ADTANKENTQRLAQMVNDFEILK
Fut8Acephalotes          LIISMIHIFKSN----------------SLSSRD---ADTANKENTQRLTQMVNDFEILK
FUT8Aechinatior          LIISMIHIFKSN----------------SLSSRD---ADTANKENTQRLAQMVNDFEILK
Fut8Phumanus             LLFAARHLSSLK----------------SGYGIGQ---NAISEGNKNRLEAALNKLHVLQ
Fut8Smaritima            ILLMTGPILHSN---------------------------ENDEHITRRLTKAMNELEVLK
Fut8Dpulex               LILLTRPLLNNP----------------Q---------SDVSSDVLQRLSKAVSELESLK
Fut8Apisum               LLVTVKLVRQQD---------------------------TNDGDSSQRLARALRELDKLH
Fut8Tcastaneum           LVFTALPMLGPH----------------I---------PSGETKTLERLKRALTDLEALR
Fut8Dmojavensis          VYVFVLKLTNSQ----------------AQNQSHQTISASDNEHNARRINQALQLLEHTE
Fut8Dpseudoobscura       VYVFVVKLTSTQ----------------GQQA------TGENEISARRISQALQLLEHTK
Fut8Dananassae           VYVFVVKLTNTQ----------------GQQA------AGESELNARRINQALQMLEHTR
Fut8Dmelanogaster        VYVFVVKLTNTQ----------------GQQA------AGESELNARRISQALQMLEHTR
Fut8Agambiae             VLIFYSKLNTSA----------------ASSS-----SSDGGDDSLKRLERAVQQLERSK
Fut8Aaegypti             VLVFYSKLNTGS----------------S----------SDSDDAIRRLNQALTYLEKSK
Fut8Cpipiens             VLIFYSKLNTGG----------------A----------NDAEDAFRRLNQALSYLEKSK
```

i/l

```
Fut8Cjaponica          2 EQNEDLKSLLDKERT--ERNDQHRKIL--------EQSESHQE-P-------------VV
Fut8Celegans           2 EQNEDLKSILEKERQ--ERNDQHKKIM--------EQSHQL---P-------------PN
Fut8Cbrenneri          2 EQNENLKDILENERR--ERNEQHRKIM--------EQTHQV---P-------------VN
Fut8Cremanei           2 EQNEKLKSIMENERR--ERNDQHRKIL--------EQSHQV---P-------------AN
Fut8Asuum                EQNEELMRLLEEREV--VAAKEAQLQQ--------NQ---L-----L-----------FA
Fut8Lloa                 HENEKLHKLLKGEKI--QPQIKK-LTV--------KKQN-DNILPIKIS-------DEFE
Fut8Bmalayi              HENEMLRELLKDEKI--EIVKRKKLIM--------KKQNDSNATRIKIN-------NKFG
Fut8Wbancrofti           HENEMLRELLKDEKI--QL-KRKKLIM--------KKQNDSNTAHIKIS-------NKFE
Fut8Tspiralis          2 QQSDSIQQAFDV--LQKQC-------------------PQLVDNR-----------NFFP
Fut8Turticae             AQNEELRNLLQN--YIPLGLQL-------------------NKASDLLKDG---------
Fut8Hmagnipapillata      RENEKLRSENIPTTLKKFKNKIANSKLSQNEENNKTLLNELANAYIQVK-----------
Fut8Nvectensis           TQNQRLKQM--VSELESQKESLE---------------SQILSKEKKIQ-----------
Fut8Moccidentalis        RQNTELRLVIQA--LNGGK-----------------------------------------
Fut8CintestinalisA     2 LQQNEYKAVLHL--A---------------DDVISRLKREAATTTT-------TSHTRLS
Fut8Lsalmonis            DDNIRLTNIVKD--YQIKEGGDSFH----------PVKDKLKESH---------------
Fut8SpurpuratusA       2 DSTQED--------L--VASSSNNG---ANDGGVDYMRNKLKDAEEIISRMEKESKGKIQ
Fut8Cteleta              HQNQELHELAEQ--LKQIQNNPVLGATADPTKEIQALKVQLAEARKQV-----------H
Fut8SkowalevskiiA      2 DQNEKLRKQANE--LREIALLRGDSEM-SKDETVKFMQQQLESAQNQI-----------A
Fut8Bfloridae          0 QQNEELRRMADE--LRYGVQMS----Q--DVDRVSLLQEQLAKCESDA-----------N
Fut8Xtropicalis        2 QQNEDLRRMAES--LRIPEGPIEQGA---AAGRIRALEEQLLKAKEQI-----------E
Fut8Rnorvegicus        2 QQNEDLRRMAES--LRIPEGPIDQGT---ATGRVRVLEEQLVKAKEQI-----------E
Fut8Hsapiens           2 QQNEDLRRMAES--LRIPEGPIDQGP---AIGRVRVLEEQLVKAKEQI-----------E
Fut8Btaurus            2 QQNEDLRRMAES--LRIPEGPIDQGP---ASGRIRALEEQLVKAKEQI-----------E
Fut8Acarolinensis      2 QQNEDLRRMAES--LRIPEGPVDQGP---AVGRVHALEEQLIKAKEQM-----------E
Fut8Ggallus            2 QQNEDLRRMAES--LRIPDGPIDQGP---AAGKVHALEEQLLKAKEQI-----------E
Fut8Mgallopavo         2 QQNEDLRRMAES--LRIPDGPIDQGP---AAGKVHALEEQLLKAKEQI-----------E
Fut8Tguttata           2 QQNEDLRRMAES--LRIPDGPIDQGP---AAGKVHALEEQLLKAKEQI-----------E
Fut8DrerioB            2 QQNEDLRRMAQS--LRIPEGQSD-GPI--SSGRLRSLEEQLSRAKQKI-----------Q
Fut8Trubipres          2 QQNEDLRRMAES--LRIPEGQADAGSL--AAGRLRSLEDQLSRAKQKI-----------H
Fut8Oniloticus         2 QQNEDLRRMAES--LRIPEGQADAGSL--AAGRLRSLEDQLTRAKQKI-----------H
Fut8Olatipes           2 QQNEDLRRMAES--LRIPEGQAEAGSL--AAGRLRSLEDQLTRAKQKI-----------H
Fut8H      melpomene   2 QQHSNLISQIRK--SSGVN-----------LN---GIDT---------------------
Fut8Dplexippus         2 QQHSNLISQIKK--SSGLN-----------VN-LNEIDA---------------------
Fut8Bmori              2 QRNSKLIAQIKK--SSGPN-----------GN-LKDLDP---------------------
Fut8Sfrugiperda        2 QQHSNLISQIKK--SSSLN-----------GN-LKDIDT---------------------
Fut8Msexta               HQHNNLIAQIRR--SSGLN-----------GN-FKDIDT---------------------
Fut8Mrotundata         2 RQNEALKNIILG---------ERSKSI--QGDHLGSIHEKLDKVS---------------
Fut8Amellifera         2 RQNEALKNIVLG---------ERSKSI--QGDHLGSIQEKLEKVS---------------
Fut8Bimpatiens         2 RQNEALKNIILG---------ERSKSI--QGDHLGSIQEKLEKVS---------------
Fut8Hsaltator          2 RQNDALKNFILG---------EGSKVM--QGDHLGSIQDRLEKAS---------------
FUT8Cfloridanus        2 KQNDALKNFILG---------EGPKSI--QGDHLGSIQDKVGKVS---------------
Fut8Sinvicta           2 KQNDALKNFILG---------EGAKSI--QGDHLGSIQDKLEKAS---------------
Fut8Acephalotes        0 KQNDALKNFILG--YANIISREGSKSI--QGDHLGSVQDRLEKAS---------------
FUT8Aechinatior          KQNDALKNFILG---------EGSKSI--QGDHLGSVQDRLEKAS---------------
Fut8Phumanus           2 QHSEHLKAVFET--LNNAEENDSVN------EILKSAEK-V-------------------
Fut8Smaritima          2 RQNDELRAILLE--FKIVDVDKSKD------QLLDDLHEKLNKAN-----------ELLH
Fut8Dpulex               VRNQELQWILTN--FSHEAQSGKIK-----EGVVERLRSTLED-K---------------
Fut8Apisum             2 KSNAELNALVLD--LNYNPRIDNKK------ILLSY----------------------FQ
Fut8Tcastaneum         2 KQNNELQEIFKD--INVDSLRGDQKEA--IENFQYRLTK---------------AEHTFS
Fut8Dmojavensis        2 RRNEELKLLIDE--LMSEQLDKQSA-----MKLIQRLENDSHNAKQSMG-----DDTLFE
Fut8Dpseudoobscura     2 QRNEELKQLIDE--LMSDQLDKQSA-----LKLVQKLENDPLNPKQGLESLGAEPEAFFD
Fut8Dananassae         2 QRNEELKQLIDE--LMSDQLDKQSA-----LKLVQRLENDAQNTKNSADGIGPEPESLFE
Fut8Dmelanogaster      2 QRNEELKQLIDE--LMSDQLDKQSA-----MKLVQRLENDALNPKLAPEVAGPEPESMFE
Fut8Agambiae             QVDQELRLLVDE--YLADAGTSADR----KQHFIDELGS-----K-------------LE
Fut8Aaegypti             QVDQELRGLIDE--YLSDQ--SYNR----KQKFVDELGN-----K-------------LQ
Fut8Cpipiens             QVDQELRTLIDE--YISDT--SYNR----KQKFVDELGN-----K-------------LQ
```

i2l

```
Fut8Cjaponica            F-------------------GPQ---TT--ARPI--KVIQSDVLGSVEQEVHKRMLDDRI
Fut8Celegans             P-------------------ENP---SLPKPEPVKEIISKPSILGPVQQEVQKRMLDDRI
Fut8Cbrenneri            S-------------------DSQ---SLPKPEPIKQIILKSSKLGSVEQEVQKRMLDDRI
Fut8Cremanei             P-------------------ESQ---SIPKPEPVKKLVAKPNKLGTIEQEVQKRMLDDRI
Fut8Asuum              0 P-------------------KAQ---SAV--PSSIAEKSTPRDLFTKEHEIARRDLDNSI
Fut8Lloa               0 K-------------------RKQ---QWIDREKSETLLLLPSNGFSMKHE----------
Fut8Bmalayi            0 K-------------------G-T---KRIERGKSEILNLLQSNGFSMKHEIARRELHNSI
Fut8Wbancrofti         0 K-------------------G-T---KRINRGKSETLSLLPSNGFSMKHEIARRELHNSI
Fut8Tspiralis            R------------------------PRVTAPEQNNHGQTQQQRRISPSHELIYQRLHLEL
Fut8Turticae             ----GLPGEANLPIPASPVSALAPLSSSPSSSSSSISLNLFASVPNLKYEQARRRVSYSY
Fut8Hmagnipapillata      -------------------------EL-----KKQLQLKESVPLHSLNYEVLSRRIFNQI
Fut8Nvectensis         0 -------------------------TL-----KR---FSD--KKPQVIHEKYRRRAENEV
Fut8Moccidentalis        -----------------------PI-----------PQQELRIAPPTDYEVHRRAAENDV
Fut8CintestinalisA     1 T-------------------STETIPT--------TTETQRPASHNLAQDPYIRKLLNDA
Fut8Lsalmonis            ---------------------------------DSSSSSETLDGPSTAYENTRRRIKHDV
Fut8SpurpuratusA       1 D-------------------------TRQQGSDSDSNQQYCGSEPSNAYEKIRRKVDNGV
Fut8Cteleta              N-------------------------LQK----------RSNAPPSLAQEELRRKVETGV
Fut8SkowalevskiiA      1 D-------------------------LSQAVGKNTKSDVSLHSGPSPDHEKLRRRIDNGV
Fut8Bfloridae          1 S-------------------------NKQQ------APVGSLDSPSKKHEQLQRKLEDGV
Fut8Xtropicalis        1 M-------------------------YKQ-------QSSNAVSGLGKDHEILRRAIENGA
Fut8Rnorvegicus        1 N-------------------------YKK-------QA---RNGLGKDHELLRRRIENGA
Fut8Hsapiens           1 N-------------------------YKK-------QT---RNGLGKDHEILRRRIENGA
Fut8Btaurus            1 N-------------------------YKK-------QT---RNGLGKDHEILRRRIENGA
Fut8Acarolinensis      1 N-------------------------YKQ-------QTG--DVGLGKEHEILRRRIENGA
Fut8Ggallus            1 N-------------------------YKK-------QTG--D-GLGKDHEILRRRIENGA
Fut8Mgallopavo         1 N-------------------------YKK-------QTV--D-GLGKDHEILRRRIENGA
Fut8Tguttata           1 N-------------------------YKK-------RTG--D-GLGKDHEILRRRIENGA
Fut8DrerioB            1 S-------------------------FQR-------LSG---EGPGREQEELRRKVENGV
Fut8Trubipres          1 S-------------------------FQK-------LTG---DGPGPTQEELRRRVENGV
Fut8Oniloticus         1 S-------------------------FQK-------LTG---DGPGPTQEELRRRVENGV
Fut8Olatipes           1 S-------------------------FQK-------LTG---DGPGPTQEELRRKAENGV
Fut8Hmelpomene           ----------------------------------SAF--HTVGGPSEEYESLRRRVYSNT
Fut8Dplexippus           ----------------------------------AEF--HNGGGPSEEYENLRRRIYSNT
Fut8Bmori                ----------------------------------AFWNLEAGLGPSEEYENLRRRIHSNT
Fut8Sfrugiperda          ----------------------------------GSLFLDGVQGPTEDYENLRRRIYSNT
Fut8Msexta               ----------------------------------GALFLNAGQGPTEEYENLRRRIHSNT
Fut8Mrotundata           ----------------------------LYDELLDTKNNIKHSVPSLEYEDLRRRVRNNV
Fut8Amellifera           ----------------------------IYEDLLDHQDNVKHGIPSSEYEELRRRIRNNV
Fut8Bimpatiens           ----------------------------LYDDLLDHQDNIKHGVPSSEYEELRRMIRNNI
Fut8Hsaltator            ----------------------------AYYDLAEQPDRLKHGVPPLEYEELRRRLRNDI
FUT8Cfloridanus          ----------------------------AYYDLIEHQDGAQHGVPSLEHEELRRRLKNDI
Fut8Sinvicta             ----------------------------VYYDLIDHQDGSKHGVPSSEYEELRRRLRNDI
Fut8Acephalotes          ----------------------------VYYDLIDHQDGSKHGIPSLEYEELRRRLRNDI
FUT8Aechinatior          ----------------------------VYYDLIDHQDGSKHGVPSLEYEELRRRLRNDI
Fut8Phumanus             -----------------------VTPF-----EEKIDVKSLYNSPSLGYETLRKRIQDDA
Fut8Smaritima          1 --------------------------------KAESGSTNTVCDPSAQFENVRRRIINQV
Fut8Dpulex               ----------------------IRLP------INFNGLEKKSSGPSKEYEVRRRAIYRGV
Fut8Apisum               -----------------------------------NSKQSGLNGPSEEYELSRRRIFSNT
Fut8Tcastaneum           K------------------------------NQ--VGYVSAKEEPNSEYELLRRRIYSNT
Fut8Dmojavensis          VIPADAAGQDQQHQQQQP----QPQPQQLGADAAGQLPGQPGIEPSLEYELTRRRIQTNI
Fut8Dpseudoobscura       SAPADLRGWNNAEGGNDPLDGAAAQPDLLS------GDDLGHGEPSMEYEFTRRRIQTNI
Fut8Dananassae           SAPADLRSWNNVVEAAPN-D------IV-D------VGEHGEIEPSLEYEFTRRRIQTNI
Fut8Dmelanogaster        SAPADLRGWNNVAEGAPN-D------LEAG------VPDHGEFEPSLEYEFTRRRIQTNI
Fut8Agambiae             QDATGSAGWF--------------------------AVGRPRNGPSLEYERLRRRVETNT
Fut8Aaegypti             IQGAGA--------------------------------GRFKGVPSLEYEQLRRRVYSNA
Fut8Cpipiens             IQGVGA--------------------------------GHFKGVPSLEYEQLRRRVYSNT
                                                                          :
```

```
Fut8Cjaponica          0 REMFYLLHSQNIENTTK-------------------ILIENQMTSLMALSGKLEKLDGSE
Fut8Celegans             REMFYLLHSQTIENSTK-------------------ILLETQMISLMGLSAQLEKLEGSE
Fut8Cbrenneri            REMFYLLHSQTIENSTK-------------------TLLENQMISLMGLSAKLEKLEGSE
Fut8Cremanei             REMFYLVHSQSIENSTK-------------------ALIENQMISLMALSAKLEKLEGSE
Fut8Asuum              0 RELFFYLNSQFEK--NDGL--------------PFARHAMNQVLSLLGQSAAFSDIDGAE
Fut8Lloa               0 ------------------------------------------LLSLLGQAAAFKNIDLAE
Fut8Bmalayi              WELYYYLDSQLISTHNNNS--------------KFINHIRNQLLSLLGQAAAFKNIDSAE
Fut8Wbancrofti         0 WELYYYLNSQLISTHNNNS--------------KFINHIRNQLLSLLGQAAAFKNIDSAE
Fut8Tspiralis          0 TELGFAIRGVFEGLLKEEKQD-----DAHQAIAKAYQRLKRQHQKLITQSLKLGEADDRL
Fut8Turticae           1 NELWFFLNDHFKHN------------------VSLLKFVNEHRASFMYDLESLSDR--DY
Fut8Hmagnipapillata      REMWFLISSQVKRMKNIVPKP------GYEDVLNFLDTYRELNQITERDFEELVSMDSVS
Fut8Nvectensis           REIFYYLSAEITKINKKIPQA------K-KQLTHMLKMSETLHQMLTNDLEKMNSLDGKS
Fut8Moccidentalis        RELWFFARAQLDKLLKEAKGA---D---RDVFKRLRQGIYERQLSALVNLDKMTRRDGYE
Fut8CintestinalisA     2 HELQLTFRARFGSPSNES------------DPNYYVQWMIDQQNALINNIEEFRDQ--NL
Fut8Lsalmonis            REMWNFINVKLKFFEHRLKS---QNEELSSKLKETREVFFSFYNAVLIDMDTLAEVDGHA
Fut8SpurpuratusA       2 TEIWYYMRSQLNQLKKSS-----GDQKTQVKIDQILENGGEQQRIKPKL-TKKALSGDFG
Fut8Cteleta            2 NEMWYLVRSQLGQLRDEVMQ--NQAAQASLRVQGLMEDAADHRRVLLTDLSNLSRANGMD
Fut8SkowalevskiiA      2 TEIWYYIKSKLNKIKKTS-----DKDQIDEQIDEILVDGGDQQRSILTDIYLLGVSGGID
Fut8Bfloridae          2 RELWFLVKAQLKLMKSDS-----QASSVKDRIDNLLTDVGDHQRTILADAQQLRDADGGL
Fut8Xtropicalis        2 KEFWYFVQSEVKKLKHLD------RNELQRHVDEIIIDMGHQQRSVMTDLYYLSQTDGAG
Fut8Rnorvegicus        2 KELWFFLQSELKKLKHLE------GNELQRHADEILLDLGHHERSIMTDLYYLSQTDGAG
Fut8Hsapiens           2 KELWFFLQSELKKLKNLE------GNELQRHADEFLLDLGHHERSIMTDLYYLSQTDGAG
Fut8Btaurus            2 KELWFFLQSELKKLKNLE------GNELQRHADEFLSDLGHHERSIMTDLYYLSQTDGAG
Fut8Acarolinensis      2 KELWFFLQSELKKLKSLE------GSELQRHTDELLSDLGDHERSIMTDLYYLSQTDGAG
Fut8Ggallus            2 KELWFFLQSELKKLKNLE------GSELQRRIDEFLSDLGHQERSIMTDLYYLSQTDGAG
Fut8Mgallopavo         2 KELWFFLQSELKKLKNLE------GSELQRRIDEFLSDLGHQERSIMTDLYYLSQTDGAG
Fut8Tguttata           2 KELWFFLQSELKKLKNLE------GSELQTRIDEFLSDLGHQERSIMTDLYYLSQTDGAG
Fut8DrerioB            2 RELWYFIHSEIQKLSHVN------PTDRQKQTDALLQDLGHQQRAIMTDLHELAQADGAG
Fut8Trubipres          2 KEFWYFVRSEVKKLVKVD------PNERQKYAETLLEDLGHQERSIMTDLYYLSQADGVG
Fut8Oniloticus         2 KEFWYFVRSEVKKLANVE------PSERQKYADTLLQDLGHQERSIMTDLYYLSQADGVG
Fut8Olatipes           2 KEFWYFVRNEVKKLANTE------LSERQKYADTLLQDLGHQERSIMTDLYYLSQADGVG
Fut8Hmelpomene         2 KELWYFVNHELSKLVNDDVQ--------TDKIKSILDQVADRKRSLLSDQEKLPELDGYH
Fut8Dplexippus         2 KEIWYFINHELTKLVNDDVQ--------PEKVQAILDQVADRKRSLLSDQEKLPKLDGYE
Fut8Bmori              2 KEFWYYANHELGKLMHESDK--------EAKIRAILEQLSDRKSTLLSDQEKLSEMDGYH
Fut8Sfrugiperda        2 KELWYYVHHELTKVIQEDNK--------AEKIQTILDQIGERKRSLLSDQQSLPELDGYN
Fut8Msexta               KELWYYVSHELGKLIHEENK--------EEKIQAILDQVADRKRSLISDEEKLPELDGYH
Fut8Mrotundata         2 QELWYYISAELSKLKKTIDKPSYDQKE--KRIQDVLRNVWDHKRSLIIDIDRLKKADGYD
Fut8Amellifera         2 QEMWYYINAELSKFRKGIDKLNYDQRE--KRIQDTLKNTWDHKKSLTIDIDRLTKADGYD
Fut8Bimpatiens         2 QETWYYINAELSKFRKTIDKLNYEQRE--KKIQDTLKNVWDHKKSLIIDIDKLMKADGYD
Fut8Hsaltator          2 QELWYYIAAELNKFKKHIDDFTHDQKEKEKDIQDVLKNTWEHKKSLIMNVDKLTKADGYN
FUT8Cfloridanus        2 QETWYYISAELNKFKKHIDEFTYDQRE--REIQDVLKNVWEHKKSLLTNIDKLKKVDGYE
Fut8Sinvicta           2 QEMWYYVSAELNKFKKHIDEFTYDQRE--KEIQDVMKNAWEHKKSLIINIDKLKKADGYN
Fut8Acephalotes        2 QEMWYYINAELNKFKKHIDEFTYDQRE--KEIQDVIKNAWEHKKSLIINIDKLKKSDGYD
FUT8Aechinatior        2 QEMWYYINAELNKFKKHIDEFTYDQRE--KEIQDVIKNAWEHKKSLIINIDKLKKSDGYD
Fut8Phumanus           2 QEFWFYISSQLEKLKGDIK---RTSPELTEKINNILKMTAEHKLSLMKDINGLSKEDGFS
Fut8Smaritima            NEMWLYTKNELKVLKTKMDDD-KT----KIKMDKMIRLETQYKRVILNDLATLSNIDGLG
Fut8Dpulex             2 QENWYFVQQELEKLKKSGRD--QSAPEISNLIQEILNSGKEHEIILLNDIQELSNMENHD
Fut8Apisum               KELWYYVNSELQSLVKEDDS------VRVEHISKIKNIIGEHYRSLLKDVSSLADVDGHA
Fut8Tcastaneum           KEFWYFIHSGLLDLEKKAH---ESAPEIVDSVKYLLSLGVEHKRSLLHDIDQLAEVDGYA
Fut8Dmojavensis          AEIWNYFSSELGKLRKAVG---PGNSELEESIQQVLLQGADHKRSLLSDMEQLRRVDGYE
Fut8Dpseudoobscura       GEIWNFFSSELAKISKVVAS--KGPKELEESINQVLLQGAEHKRSLLSDMDHMRRSDGYE
Fut8Dananassae           GEIWNFFSSELGKVRKAVAAG-HANTDLEETINQVLLQGAEHKRSLLSDMEHLRRSDGYE
Fut8Dmelanogaster        GEIWNFFSSELGKVRKAVAAG-HASADLEESINQVLLQGAEHKRSLLSDMERMRQSDGYE
Fut8Agambiae             QELWNFLQSEVTKVQKQAL---RSAPELAKPLNAFLSLAADHKRSLLTDIERMRAADGYE
Fut8Aaegypti             QELWNLLHSETAKLAKTAR---RNTPEIYAPAENFLALAAEHKRSLLNDINQMRASDGYE
Fut8Cpipiens             QELWNFLNSEVIKAAKIGR---KNSPEIAPPLDALLSLASEHKRSLLNDINQMRASDGYE
```

i3l

```
Fut8Cjaponica           DRRKTLRSELASRIHKSIDHLQNPTSCADAKTLLCNLDKECGFGCQLHHVTYCAITAFAT
Fut8Celegans            EERFKQRTAITQRIFKSIEKLQNPKACSEAKTLVCNLDKECGFGCQLHHVTYCAITAFAT
Fut8Cbrenneri           DERIKKRSEITNRIMKSIKKLQNPKSCGEANTLVCNLDKECGFGCQLHHVTYCAITAFAT
Fut8Cremanei            DERMSQRSEITNRILKSIEKLQNPQSCGDVNTLVCNLDKECGFGCQLHHVTYCAITAFAT
Fut8Asuum             2 KWREAALATISAKIQSQLDRLQNPNDCTSARSLICHLNKGCGFGCQLHHVTYCFIVAYGT
Fut8Lloa              2 SWRANALANISAIFQNHFDKMQNPDDCKSARILTCDLNKQCGFGCQLHHVTYCFIVAYGS
Fut8Bmalayi           2 SWRANALANISAIFQNHFNKMQNPDDCKTARILTCDLNKQCGFGCQLHHVTYCFIVAYGS
Fut8Wbancrofti        2 SWRTNALANISAIFQNHFNKMQNPDDCKTARILTCDLNKQCGFGCQLHHVTYCFIVAYGS
Fut8Tspiralis         1 QLRS-----------ARLQHLGNA---------VQEQLGPCGFGCQMHHVTFCLIVSYYT
Fut8Turticae            EWRKNEFKKLTELFTSVVDKLQNPPNCATANKLVCRLNKGCGFGCQMHHVSYCMVVALAT
Fut8Hmagnipapillata     EYKDSLALELSNIVQKRIFTLQNPKNCDTAKKLVCSLNKGCGYGCQMHHVLYCFIVAYST
Fut8Nvectensis          KWQWSEHEELSRIVQERIKKLQNPKDCSTSKKLICQLNKGCGYGCQVHHVLYCFIVAFGS
Fut8Moccidentalis     2 MWRKQEAEDLQRIVLDRIRVLQNPADCSSAKKLFCNLNKGCGYGCQLHHATYCMIMAISM
Fut8CintestinalisA    0 EARGEAARKLQDLVQKRINYLQNPKDCRSNRKLVCAIHKKCGFGCQLHHLSYCMMTAYGT
Fut8Lsalmonis           AWRLQESKNLSLHVQSRLTALQNPSDCKNARKLLCNLNKGCGYGCQIHHVVYCFVSAIGA
Fut8SpurpuratusA      0 ---------DGFVVRGHIGKSENPKDCSKAKKIVCNLNKGCGYGCQVHHVAYCMIVAYAT
Fut8Cteleta           0 EWRHREHEQLTELVQTRIKYLQNPKDCSKAKKLVCNINKGCGYGCQLHHLTYCFIIAYGT
Fut8SkowalevskiiA     0 MWRENEAKELEQTVQKRLKYLQNPKDCSKAKKLVCNLNKGCGYGCQLHHVVYCLIMAYGS
Fut8Bfloridae         0 AWRQREAHALQDLVQRRLHYLQNPKDCSKAKKLLCNLNKGCGYGCQLHHVVYCFMVAYGT
Fut8Xtropicalis       0 DWREREAKDLTDLVQRRITYLQNPKDCSKAKKLVCNINKGCGYGCQLHHVVYCFMIAYGT
Fut8Rnorvegicus       0 DWREKEAKDLTELVQRRITYLQNPKDCSKARKLVCNINKGCGYGCQLHHVVYCFMIAYGT
Fut8Hsapiens          0 DWREKEAKDLTELVQRRITYLQNPKDCSKAKKLVCNINKGCGYGCQLHHVVYCFMIAYGT
Fut8Btaurus           0 DWREKEAKDLTELVQRRITYLQNPKDCSKAKKLVCNINKGCGYGCQLHHVVYCFMIAYGT
Fut8Acarolinensis     0 DWREKEAKDLTDLVQRRIIYLQNPKDCSKAKKLVCNINKGCGYGCQLHHVVYCFMIAYGT
Fut8Ggallus           0 DWREKEAKDLTDLVQRRITYLQNPKDCSKAKKLVCNINKGCGYGCQLHHVVYCFMIAYGT
Fut8Mgallopavo        0 DWREKEAKDLTDLVQRRITYLQNPKDCSKAKKLVCNINKGCGYGCQLHHVVYCFMIAYGT
Fut8Tguttata          0 DWREKEAKDLTDLVQRRITYLQNPKDCSKAKKLVCNINKGCGYGCQLHHVVYCFMIAYGT
Fut8DrerioB           0 EWRDKEARDLSILVQNRISFLQNPQDCSKARKLVCNINKGCGYGCQLHHVVYCFMIAYGT
Fut8Trubipres         0 EWRMKEAKDLSDLVQNRITYLQNPPDCSKARKLVCNINKGCGFGCQLHHVVYCFMIAYGT
Fut8Oniloticus        0 EWRMKEAKDLSDLVQNRITYLQNPPDCSKARKLVCNINKGCGYGCQLHHVVYCFMIAYGT
Fut8Olatipes          0 DWRTKEAKDLSDLVQNRITYLQNPPDCSKARKLVCNINKGCGYGCQLHHVVYCFMIAYGT
Fut8Hmelpomene        0 EWRVTEAANVSDLVQRRLKYLQNPSDCKDARKVICNLNKGCGFGCQLHHIVYCLIFAYAT
Fut8Dplexippus        0 DWRRSEASEVSDLVQKRLKYLQNPPDCRDARKVICNLNKGCGFGCQLHHIVYCLIFAYAT
Fut8Bmori             0 EWRQLEAANVSDLVQRRLHYLQNPPDCREARKLICNLNKGCGFGCQLHHIVYCLIFAYAT
Fut8Sfrugiperda       0 EWRQAEAANVSDLVQRRLHYLQNPPDCKDARKLICNLNKGCGFGCQLHHIVYCLIFAYAT
Fut8Msexta              EWRQMEAANVSDLVQRRLHYLQNPPDCREARKLICNLNKGCGFGCQLHHIVYCLIFAYAT
Fut8Mrotundata          EWRKKEAKDLSDLVQRRFKYLQNPSDCNKARKLVCSLNKGCGFGCQIHHITYCFLVAYGT  0
Fut8Amellifera          EWRKKETKELSDLVQRRFKYLQNPSDCNKARKLICSLNKGCGFGCQIHHITYCFLVAYGT  0
Fut8Bimpatiens          EWRKKEAKELSDLVQRRFRHLQNPSDCNKARKLVCSLNKGCGFGCQIHHITYCFLVAYGT  0
Fut8Hsaltator         2 EWREKEAKDLSDLVQRRFRYLQNPTDCNKAKKLVCSLNKGCGFGCQLHHITYCFMVAYGT  0
FUT8Cfloridanus       2 EWRKKEAKDLSDLVQKRFRYLQNPADCSKAKKLICSLNKGCGFGCQIHHITYCFLVAYGT  0
Fut8Sinvicta          2 EWREKEAKDLSDLVQQRFRYLQNPPDCNKAKKLICSLNKGCGFGCQIHHITYCFLVAYGT  0
Fut8Acephalotes       2 EWREKEAKDLSDLVQRRFRYLQNPTDCNKAKKLICSLNKGCGFGCQIHHITYCFLVAYGT  0
FUT8Aechinatior         EWREKEAKDLSDLVQRRFRYLQNPTDCNKAKKLICSLNKGCGFGCQIHHITYCFLVAYGT
Fut8Phumanus          0 SWRKSESASLAAELEKRLKLLQNPEDCTTAKKLICNLNKGCGFGCQIHHAAYCLIVAYAS
Fut8Smaritima         0 EWREREAKSLSDLIQRRLHYLQNPKDCKNAKKLNCNLNKGCGYGCQIHHVVYCFIVAYGS
Fut8Dpulex            0 AWRSTEARALSDLVQRRLHYLQNPADCSKARKLICNLNKSCGYGCQIHHAAYCFIMAYAT
Fut8Apisum            0 AWRQQESENLSNLVQKRLKHLQNPSDCAKARKLVCDLNKGCGYGCQLHHVVYCFIVAYAT
Fut8Tcastaneum        0 TWREKEANDLSGLVQKRFHFLQNPADCKTAKKLICSLNKGCGYGCQLHHAVYCFMVAYGT
Fut8Dmojavensis       0 AWRQQEAKDLSDLVQRRLHHLQNPSDCQNARKLVCKLNKGCGYGCQLHHVVYCFIVAYAT
Fut8Dpseudoobscura    0 TWRHKESRDLSDLVQRRLNHLQNPSDCQNARKLVCKLNKGCGYGCQLHHVVYCFIVAYAT
Fut8Dananassae        0 AWRHREAKELSDLVQRRLHHLQNPRDCQNARKLVCKLNKGCGYGCQLHHVVYCFIVAYAT
Fut8Dmelanogaster     0 AWRHKEARDLSDLVQRRLHHLQNPSDCQNARKLVCKLNKGCGYGCQLHHVVYCFIVAYAT
Fut8Agambiae            AWRHREAADLSELVQKRLTRLQNPENCSTARKLLCRLNKGCGYGCQLHHVVYCFIMAYAT
Fut8Aaegypti            FWRYKESKDLSDLVQKRLTHLQNPEDCNSARKLICRLNKGCGYGCQLHHVVYCFIMAYAT
Fut8Cpipiens            FWRYKESKDLSDLVQRRLSYLQNPEDCSTARKLVCRLNKGCGYGCQLHHVVYCFIMAYAT
                                         .    *                 **:***:**  :* : :
```

i4c

Cystein-rich domain

i4l

```
Fut8Cjaponica            QRMLILRRDGQSWKYSAR---------GWTSVFKPISKCSYDEA--VGKT------DLKP  0
Fut8Celegans           2 QRMMVLKRDGSSWKYSSH---------GWTSVFKKLSKCSFDEA--VGNT------EAKP  0
Fut8Cbrenneri          2 KRMLILKRDGSSWKYSSR---------GWTSVFEPISKCSFDEA--VGKT------ELKP  0
Fut8Cremanei           2 KRMMILKRDGSSWKYSSR---------GWTSVFEPISKCSFDDA--VGKA------EMKP  0
Fut8Asuum                NRTLILLHDGRDWNYSGK---------GWTAAFLPISRCKHADV--SKD--EGNEEEWSS  0
Fut8Lloa                 NRTLVLTDDGRTWNYATN---------GWTAAFLPITKCSFSEI--FKP--NEYADDWGT  0
Fut8Bmalayi              NRTLVLTDDGRTWNYAAN---------GWNAAFLPITKCSFSEI--FKVTLNENADDWGI  0
Fut8Wbancrofti           NRTLVLTDDGRTWNYAAN---------GWNAAFLPITKCSFSEI--FKVTARFPKD----
Fut8Tspiralis            NRTMILRGD--RWSYSSR---------GWTSVFKPLSETCTEAE-------DVPPSMFTA
Fut8Turticae             KRTMILDSN--NWRYAPNPNAHGSREPSWNLIFQPLSRTCTTAD-------GKTHKPWSE
Fut8Hmagnipapillata      GRTMIIDSS--GWRYSSK---------GWDAYFES-VTSCKDFQ-------E-A-KEWGY
Fut8Nvectensis           QRTMIIDST--GWRYSSS---------GWSGIFLQPSETCTSYT-------G-GFSNWQR
Fut8Moccidentalis        KRTLILQSK--GWRYNPR---------GYEDIFQPVSNSCIAET-------GGSRSKYPA
Fut8CintestinalisA       GRTMILVSK--LWLYNPG---------GWEEIFEPLSATCTQTF-------NFKPQVWRG  1
Fut8Lsalmonis            GRALVLKSK--GWRYNRA---------GFENVFKPLSETCQRIPEVLQGELPLSRSSWPG  1
Fut8SpurpuratusA         ERTLILDSK--GWRYARE---------GWEKFFLPLSETCLDRK-------GESSGRWGA
Fut8Cteleta              QRTFVLESR--GWRYSSE---------GWEKYFLPLSETCTDRQ-------GDSSRAWGG
Fut8SkowalevskiiA        ERTLILESK--GWRYAKD---------GWEKFFQPLSNTCVTIT-------GTSRNAWTE  1
Fut8Bfloridae            ERTMILNSQ--GWRYSSG---------GWEKVFLPLSETCTDPS-------GSSRGPWKG  1
Fut8Xtropicalis          QRTLILESQ--SWRYATG---------GWETVFKPVSETCTDRS-------GSSTGHWSG  1
Fut8Rnorvegicus          QRTLILESQ--NWRYATG---------GWETVFRPVSETCTDRS-------GLSTGHWSG  1
Fut8Hsapiens             QRTLILESQ--NWRYATG---------GWETVFRPVSETCTDRS-------GISTGHWSG  1
Fut8Btaurus              QRTLILESH--NWRYATG---------GWETVFRPVSETCTDRS-------GVSTGHWSG  1
Fut8Acarolinensis        QRTLILESQ--NWRYATG---------GWETVFRPVSETCTDRS-------GSTTGHWSG  1
Fut8Ggallus              QRTLILESQ--NWRYATG---------GWETVFRPVSETCTDRS-------GTTTGHWSG  1
Fut8Mgallopavo           QRTLILESQ--NWRYATG---------GWETVFRPVSESCTDRS-------GTTTGHWSG  1
Fut8Tguttata             QRTLILESQ--NWRYATG---------GWETVFRPVSETCTDRA-------GSSTGHWSG  1
Fut8DrerioB              QRVLILESH--NWRYAPK---------GWETVFKAVSETCTDRS-------GATTGHWSG  1
Fut8Trubipres            QRTLILESH--NWRYAPG---------GWETVFLPVSNTCTDRS-------GATTGHWSG  1
Fut8Oniloticus           QRTLILESH--NWRYAPG---------GWETVFLPVSNTCTDRS-------GATTGHWSG  1
Fut8Olatipes             QRTLILESH--NWRYAPG---------GWETVFLPVSNTCTDRS-------GASTGHWSG  1
Fut8Hmelpomene           ERTLILNSK--GWRYNNK---------GWEYVFHPISDTCVSVY-------DDKVVQWPA  1
Fut8Dplexippus           ERTLILNSK--GWRYNNK---------GWEYVFHPISDTCTTAY-------DDKVVPWPA  1
Fut8Bmori                ERTLILNSK--GWRYNTK---------GWEYVFHPISESCLSSY-------DDKVVQWPV  1
Fut8Sfrugiperda          ERTLILNSK--GWRYNNK---------GWEYVFMPISDTCTSAY-------DDKVVMWPV  1
Fut8Msexta               ERTLILNSK--GWRYNTK---------GWDYVFYPISDSCTSAY-------DDKVVQWPV
Fut8Mrotundata           ERTLIIKSK--GWRYHKD---------GWESVFKPLSDTCVSTN-------GASHANWPG
Fut8Amellifera           ERTLIIRSK--GWRYHKD---------GWESVFKPLSDTCVSTN-------GASHANWPG
Fut8Bimpatiens           ERTLIIRSK--GWRYHKD---------GWESVFKPLSETCVSTI-------GASHANWPG
Fut8Hsaltator            ERTLIIKSK--GWRYHKD---------GWESVFKPLSDTCVSTS-------GVSHSNWPG
FUT8Cfloridanus          ERTLIIKSK--GWRYQKE---------GWESVFKPLSNTCLSTT-------GVSHSNWPG
Fut8Sinvicta             ERTLIMKSK--GWRYHKD---------GWESVFKPLSDNCLSTT-------GTSHSNWPG
Fut8Acephalotes          ERTLIMKSK--GWRYHKD---------GWESVFKPLSDTCLSTT-------GTSHSNWPG
FUT8Aechinatior          ERTLIMKSK--GWRYHKD---------GWESVFKPLSDTCLSTS-------GTSHSNWPG
Fut8Phumanus             KRTLILTSK--GWRYHKS---------GWEDIFLPLSDTCRSPS-------GASRVGWPG  1
Fut8Smaritima            GRTLILNSK--GWRYNKD---------GWESVFKPVSDSCTSTM-------GVGSAMWPG
Fut8Dpulex               KRTLILNSK--KWRYHRG---------GWEKVFLPLSDTCTDPS-------GLDRSNWPG  1
Fut8Apisum               ERTLILRSK--GWRYSKG---------GWQDVFLPLSDTCLLPN-------GETTNRWPG  1
Fut8Tcastaneum           KRTLILKSK--GWRYHKA---------GWEEIFKPVSDTCVDPS-------GKSVSNWPG  1
Fut8Dmojavensis          ERTLILKSR--GWRYHKG---------GWEEVFRPVSDNCQDAG-------TAYAYNWPG
Fut8Dpseudoobscura       ERTMILKSR--GWRYHKG---------GWEEVFQPISKGCHDAG-------SANAYNWPG
Fut8Dananassae           ERTLILKSR--GWRYHKG---------GWEEVFQPVSNSCHDAG-------TANTYNWPG
Fut8Dmelanogaster        ERTLILKSR--GWRYHKG---------GWEEVFQPVSNSCHDAG-------TANTYNWPG
Fut8Agambiae             ERTLILKSK--GWRYHKA---------GWEEVFQPISDTCLDSN-------GASHASWPG
Fut8Aaegypti             ERTLVLKSK--GWRYHKA---------GWEEVFQPISDTCLSAD-------GATHASWPG
Fut8Cpipiens             ERTLILKSK--GWRYHKA---------GWEEVFQPISDTCLDAN-------GATHASWPG
                          * :::      * *              .:    *
```

i5l

```
Fut8Cjaponica           2 FTENSPARVLSLGIVDSLTIK--PEFLPQAVPQQILRNLTAIHSHPPAFFVGTFISYLMR  0
Fut8Celegans            2 FAEPSPARVVSLGIVDSLITK--PTFLPQAVPEQLLESLTSLHSHPPAFFVGTFISYLMR  0
Fut8Cbrenneri           2 FTDPSPARVVSLGIVDSLLTK--PTFLPQAVPEQLLGNLTALHSHPPAFFVGTFISYLMR  0
Fut8Cremanei            2 FADPSPERVVSLGIVDSLLTK--PAFLPQAIPEQLLSTLSSLHSHPPAFFVGTFISYLMR  0
Fut8Asuum                 VAMSARSRVVKLPIIDGLHGR--PPFLPLAFPEFLASDLLKLHSNPPVFFISQFLRYLMR  0
Fut8Lloa                  GKHYRNKRIVKLPIIDSLAFR--PPYLPLAIPQSYSDELLKLHSNPPAFFISQFIRYLMR  0
Fut8Bmalayi               GEHYKDKRIVKLPIIDSLSQR--PAYLPLAIPQLYSAELQKLHSNSPAFFISQFIRYLMR  0
Fut8Wbancrofti          0 FSHYRDKRIVKLPIIDSLSQR--PPYLPLAIPQLYSAELQKLHSNSPVFFISQFIRYLMR  0
Fut8Tspiralis           1 EGDLKKSFLVDAPIVDIIFDKEQYPYLPLSFPKELADELLQVHSYPPVWWIGQFVRYLMR  2
Fut8Turticae            0 GV---TAQVVDLPIIDSLRPR--MDYLPRAIPEQISEKLITLHGSPIVWFMGQILGFIMR
Fut8Hmagnipapillata       DH--ASHQVVHLPIVDALFPR--PSQMPQAVPKDLFDQIRMFHGHPFVWWIGQFCKFLFK
Fut8Nvectensis            -N--NDAQNVILPIVDSLYPR--PPYMPMAVPEDLADRLSRLHGHPFVWWIGQFAKYLFR
Fut8Moccidentalis       0 SG--DEVVNLEMPIIDGLIQK--PDCLPLAVPKALVPRLSQLHANPALWWISQIVGFLQR
Fut8CintestinalisA        ELESANELEVMLPAVDQLPKA--PRFAPLGIPEDLADELDQFHGSPGVWWVGQFIKYIFR
Fut8Lsalmonis             KE---DTVNVDIPIVDSVNPR--PKHLPPSIPKEIASRIIRLHGDPIVWWISEVLRYILR
Fut8SpurpuratusA          PSNIEHLQVVELPIVDGLHPR--PEFLPLAIPEDISQRLMRIHGHPIVWWMGQIMTYIQR
Fut8Cteleta             0 KKRLTYVQVVDLPIVDSLHPR--PPFMPQAIPADLAPRLMRMHGHPFVWWIAQFLTYLTR
Fut8SkowalevskiiA         ESRIKDVQVVDLPIVDGLHPR--PNYLPLAIPEDLSERLLRLHGQPAVWWIAQFIKYVTR
Fut8Bfloridae             EK-SKDTQVVEMPIVDSIYPR--PPYLPLGIPEDLSDRLLRLHGNPSVWFIAQFVLYLQR
Fut8Xtropicalis           EANDKNVQVVELPIVDSLHPR--PPYLPLGVPEDLADRLIRLHGDPAVWWVSQFVKYLIR
Fut8Rnorvegicus           EVNDKNIQVVELPIVDSLHPR--PPYLPLAVPEDLADRLVRVHGDPAVWWVSQFVKYLIR
Fut8Hsapiens              EVKDKNVQVVELPIVDSLHPR--PPYLPLAVPEDLADRLVRVHGDPAVWWVSQFVKYLIR
Fut8Btaurus               EIKDKNVQVVELPIVDSLHPR--PPYLPLAVPEDLADRLVRVHGDPAVWWVSQFVKYLIR
Fut8Acarolinensis         ETNDKDVQVIELPIVDSLHPR--PPYLPLAIPEDLADRLIRVHGDPAVWWVSQFVKYLIR
Fut8Ggallus               ETNDKDVQVVELPIVDSLHPR--PPYLPLAVPEDLADRLIRVHGDPAVWWVSQFVKYLIR
Fut8Mgallopavo            EANDKDVQVVELPIVDSLHPR--PPYLPLAVPEDLADRLIRVHGDPAVWWVSQFVKYLIR
Fut8Tguttata              EANDKDVQVVELPIVDSLHPR--PPYLPLAVPEDLADRLIRVHGDPAVWWVSQFVKYLIR
Fut8DrerioB               EAHDRDVQVVELPIVDSLHPR--PPYLPLAVPEDLAPRLQRLHGDPSVWWVSQFVKFLVR
Fut8Trubipres             EAHDKDVQVVELPIVDSLHPR--PPYLPLAIPEDLAPRLQRLHGDPSVWWVSQFVKYLVR
Fut8Oniloticus            EAHDKDVQVVELPIVDSLHPR--PPYLPLAIPEDLAPRLQRLHGDPSVWWVSQFVKYLVR
Fut8Olatipes              EAHDKDVQVVELPIVDSLHPR--PPYLPLAIPEDLAPRLHRLHGDPSVWWVSQFVKYLVR
Fut8Hmelpomene            SY---DAKVVSLPFIDSISQK--PKFLPLAIPSDLAHRIVRFNGDPASWWIGQMLKYVLK  2
Fut8Dplexippus            SY---DAKVVSLPFIDSVSQK--PKFLPLAVPSDLAHRIVRFNGDPSSWWIGQMLKYVLK  2
Fut8Bmori                 SY---DAKVVSLPFIDSISQK--PKFLPLAVPKDLAHRIVRFNGDPSSWWVGQMLKFVLK  2
Fut8Sfrugiperda           TY---DAKVLALTFIDSISQK--PKFLPLAIPKDLAHRITRFNGDPSSWWIGQMLKYILK  2
Fut8Msexta                LY---DAKVVSLPFIDSISQK--PKFLPLAIPKDLAPRIVRFNGDPSSWWIGQMLKYILK
Fut8Mrotundata            DA---SKQVISLPIVDNVYPK--PRYQPPSVPEDLAPRLEKLHGHPLVWWVGQVLKYLMR
Fut8Amellifera            DS---SKQVISLPIVDNVYPK--PRYQPPSVPADLASRLEKLHGHPLVWWVGQVLKYLMR
Fut8Bimpatiens            DS---SKQVISLPIVDNVYPK--PRFQPPSVPADLASRLEKLHGHPLVWWVGQVLKYLMR
Fut8Hsaltator             DP---TKQVISLPIVDNVYPK--PKYQAPSVPADLAARLEKIHGHPLVWWVGQVLKYLMR
FUT8Cfloridanus           DP---SKQVISLPIVDNVYPK--PRFQPPSVPADLAPRLEKIHGHPLVWWVGQVLKYLMR
Fut8Sinvicta              DP---SKQVISLPIVDNVYPK--PRFQPPSVPADLAPRLEKIHGHPLVWWVGQVLKYLMR
Fut8Acephalotes           DP---SKQVISLPIVDNVYPK--PRFQPPSVPADLAPRLEKIHGHPLVWWVGQVLKYLMR
FUT8Aechinatior           DP---SKQVISLPIVDNVYPK--PRFQPPSVPADLAPRLEKIHGHPLVWWVGQVLKYLMR
Fut8Phumanus              QS---DSQVVVLPIIDTLFPR--PEFLPPAIPKDLAPRLIKLHSQPLAWWIGQMLRYLMR
Fut8Smaritima             YG---DAQVLDLPIIDSISPR--PDYLPLAIPRDLAARMQRLHGDPIVWWVGQFLKYLLR
Fut8Dpulex                TN---ETQVIELPIVDMLSPR--PPFLPLAIPRDLSERMIRLHGDPQVWWIGQFMKYLLR
Fut8Apisum                HR---NTQVITLPIIDSINPR--PPFLPLALPEDLAPRLNVLHGDPVVWWIGQFLKYMLR
Fut8Tcastaneum            NS---ETQVVNLPIIDSLSPR--PPFLPLAIPEDLAPRLTRLHGDPIVWWVGQILKFLLR
Fut8Dmojavensis           KP---NTQVLVLPIIDSLMPR--PPYLPLAVPEDLAPRLKRLHGDPIVWWVGQFLKYLLR
Fut8Dpseudoobscura        KP---NTQVLVLPIIDSLMPR--PPYLPLAVPEDLAPRLKRLHGDPIVWWVGQFLKYLLR
Fut8Dananassae            KP---NTQVLVLPIIDSLMPR--PPYLPLAVPEDLAPRLKRLHGDPIVWWVGQFLKYLLR
Fut8Dmelanogaster         KP---NTQVLVLPIIDSLMPR--PPYLPLAVPEDLAPRLKRLHGDPIVWWVGQFLKYLLR
Fut8Agambiae              QS---NTQVLTLPIIDSLNPR--PPYLPLAIPADLAPRLMKLHGDPIVWWIGQFLKYLLK
Fut8Aaegypti              TP---ETQVLTVPIIDSLNPR--PPYLPLAIPADLAPRLIKLHGDPIVWWIGQFLKYLLK
Fut8Cpipiens              TT---ETQVLTVPIIDSLNPR--PPYLPLAIPADLAPRLMKLHGDPIVWWIGQFLKYLLK
                                   :    :* :           ..*      :  ...    :::. .  :: :
```

i6l

```
Fut8Cjaponica          FNVETIEKLEAAVQS--MKLSRGPIVGLQIRRTDKVGTEAAFHALKEYMEWTEIWFKVEE
Fut8Celegans           FNSATQEKLDKALKS--IPLDKGPIVGLQIRRTDKVGTEAAFHALKEYMEWTEIWFKVEE
Fut8Cbrenneri          FNSDTKEKLEEAVKA--IPFDKGPVVGLQIRRTDKVGTEAAFHGLKEYMEWTEIWFKVEE
Fut8Cremanei           FNAETKEKLEAALKA--IPFSEGPVVGLQIRRTDKVGTEAAFHALKEYMEWAEIWFKIEE
Fut8Asuum            1 PNDELAKRITEAASK--VPFNKGPVVGLQIRRTDKVGTEAAFHALPEYMKWTEYWFQIEE
Fut8Lloa             1 PSAVFAKEIDQAVKK--VPFDKGPIVGLQIRRTDKIHTEASFHDLDEYIKWAEDWFKIEE
Fut8Bmalayi          1 PSAVFAKEIDLAVKK--VPFDKGPIVGLQIRRTDKIHTEASFHDLDEYMKWAEDWFQIEE
Fut8Wbancrofti       1 PSAVFAKEIDLAVKK--VPFDKGPIVGLQIRRTDKIHTEASFHDLDEYMKWAEDWFQIEE
Fut8Tspiralis        2 FVDSVKLDLDEKLET--YGF-RHPIVGIHVRRTDKLLSEASFHSVKEYMEEVENWYDVQM
Fut8Turticae           PSQEMKKFLENAKKR--FKF-RSPIIGIHVRRTDKVGTEASYHSLTEYMKYAEQYFQNLE
Fut8Hmagnipapillata  2 YKPDLQREITEKKKS--LGF-KSPIVGVQIRRTDKINLEAAYHDIDEYMYWVDLYYKKLA
Fut8Nvectensis       2 YNANVQKEINEKRER--MGF-KSPIVGIQVRRTDKINTEAARHEVEEYMYWVQLYYSRLG
Fut8Moccidentalis    0 PQRGTSDFLKEVRDH--HGI-KGPYAGIHVRRTDKIGTEADYHGIDEYMSWVAEYFDKYE  2
Fut8CintestinalisA   2 PRPWLRKELDGYEVT--VDY-STPIVGVHVRRTDKIGKEASFHAIDEYMKHVDDWFDRYD
Fut8Lsalmonis          PQEDLQVLLNSTEEDKLPRD-KSPYVGIHVRRTDKVGTEAAFHPVEEYMEYVEERFKYLD
Fut8SpurpuratusA     2 PQPALQEDIDKMSQA--LGF-TNPIVGLHVRRTDKVGTEAAFHGIEEYMFHAEEFYLRLE
Fut8Cteleta          2 LQPAFQRDIDDVSHS--LGF-KGPIVGVHVRRTDKVGTEAAFHGLDEYMIYVEEYFNLLE
Fut8SkowalevskiiA    2 PQPVLQSFLDETRNK--VKF-ENPIVGVHVRRTDKVGTEAAFHNIDEYMVHVEEYYKRLS
Fut8Bfloridae        2 YQPYLQQDIEETAQK--LGF-KHPIVGIHVRRTDKINTEAAYHSIAEYMEWVEEYYQLLQ
Fut8Xtropicalis      2 PQPWLEKEIEESTKK--LGF-KHPVIGVHVRRTDKVGTEAAFHPIEEYMVHVEEHFQLLA
Fut8Rnorvegicus      2 PQPWLEKEIEEATKK--LGF-KHPVIGVHVRRTDKVGTEAAFHPIEEYMVHVEEHFQLLA
Fut8Hsapiens         2 PQPWLEKEIEEATKK--LGF-KHPVIGVHVRRTDKVGTEAAFHPIEEYMVHVEEHFQLLA
Fut8Btaurus          2 PQPWLEKEIEEATKK--LGF-KHPVIGVHVRRTDKVGTEAAFHPIEEYMVHVEEHFQLLA
Fut8Acarolinensis    2 PQPWLEKEIEEATRK--LGF-KHPVIGIHVRRTDKVGTEAAFHPIEEYMIHVEERFQLLA
Fut8Ggallus          2 PQPWLEKEIEEATRK--LGF-KHPVIGVHVRRTDKVGTEAAFHPIEEYMVHVEERFELLA
Fut8Mgallopavo       2 PQPWLEKEIEEATRK--LGF-KHPVIGVHVRRTDKVGTEAAFHPIEEYMVHVEERFELLA
Fut8Tguttata         2 PQPWLEKEIEEATRK--LGF-KHPVIGVHVRRTDKVGTEAAFHPIEEYMVHVEERFELLA
Fut8DrerioB          2 PQAWLEKEIQETCLK--LGF-KHPIIGVHVRRTDKVGTEAAFHPIEEYMVHVEEQFQSMA
Fut8Trubipres        2 PQAWLEKEIQQSTAK--LGF-KHPIVGVHVRRTDKVGTEAAFHPIEEYMIHVEEQFQLLA
Fut8Oniloticus       2 PQAWLEKEIQQTTAK--LGF-KHPIIGVHVRRTDKVGTEAAFHPIEEYMLHVEEQFQLLA
Fut8Olatipes         2 PQGWLEKEIQQSTAK--LGF-KHPIIGVHVRRTDKVGTEAAFHPIEEYMLHVEEQFQLLA
Fut8Hmelpomene       2 PKEETQKAINETIAK--LNF-KNPIVGVHVRRTDKVGTEAAFHHLHEYMQHVQHYYEQLQ
Fut8Dplexippus       2 PRAAMQKAINETIAK--MNF-KNPIVGVHIRRTDKVGTEAAFHHIDEYMVHVKEYYRTLE
Fut8Bmori            2 PRLPMQKAINDTIAK--MNF-KSPIVGVHIRRTDKVGTEAAFHHIHEYMAHVKDYYDQLE
Fut8Sfrugiperda      2 PRQAMQKAINDTIAK--MNF-KKPIVGVHIRRTDKVGTEAAFHHIHEYMVHVKDYYDQLE
Fut8Msexta             PRVAMQKAINETIAK--MNF-KTPIVGVHIRRTDKVGTEAAFHHIHEYMAHVKDYYDQLE
Fut8Mrotundata         PQEHVTKTLNYAKDR--LGF-KKPIVGIHVRRTDKVGTEAAYHDIDEYMVKVEQYFDELE
Fut8Amellifera         PQEHVKKMLDDAKER--LGF-KKPIVGIHVRRTDKVGTEAAYHDIDEYMIKVDQYFDELE
Fut8Bimpatiens         PQEHVKRTLENAKEK--LGF-KKPIVGVHVRRTDKVGTEAAYHDIDEYMVKVEQYFDELE
Fut8Hsaltator          PQDHVKKTLEKAKEK--LGF-KKPVVGVHVRRTDKVGTEAAYHDIDEYMTKVEQYFDELE
FUT8Cfloridanus        PQDHVKRTLEKARER--LGF-KKPIVGVHVRRTDKVGTEAAYHDIDEYMSKVEQYFDELE
Fut8Sinvicta           PQDHVKRTLEKAKER--LGF-KKPIVGIHVRRTDKVGTEAAYHDIDEYMTKVEQYFDEFE
Fut8Acephalotes        PQDHVKRTLEKAKER--LGF-KKPIVGVHVRRTDKVGTEAAYHDIDEYMIKVEQYFDELE
FUT8Aechinatior        PQDHVRRTLEKAKER--LGF-KKPIVGIHVRRTDKVGTEAAYHDIDEYMIKVEQYFDELE
Fut8Phumanus         2 PQPSTEKFFNESINK--LNF-KKPIVGVHIRRTDKIGTEADFHSIEEYMKHVGDWFDQQI
Fut8Smaritima        2 PQPNLQANIDEMTDK--LGF-KKPIVGIHVRRTDKVGTEAAFHKLHEYMLHAGEFYQQLA
Fut8Dpulex           2 YQPETQKMLDQAKEK--MKF-ESPVVGVHVRRTDKVGTEAAFHSIDEYMLYVADFFDKLE
Fut8Apisum           2 PQPATSNKLDEYGKK--VKF-QKPIVGVHIRRTDKVGTEAAFHKLDEYMVHVEQYYKYKE
Fut8Tcastaneum       2 PQPQTAAVMQETMTH--MGF-KRPIVGVHVRRTDKVGTEAAYHGIEEYMTAVDEYYKELE
Fut8Dmojavensis      2 PQTETRDFLNSGMRK--LGW-ERPIVGVHVRRTDKVGTEAAFHGIDEYMTHVEDYYRTLE
Fut8Dpseudoobscura   2 PQQPTREFLFAGMRN--LGW-ERPIVGVHVRRTDKVGTEAACHSIGEYMTHVEDYYRTLE
Fut8Dananassae       2 PQTTTRDFLTAGMRN--LGW-ERPIVGVHVRRTDKVGTEAACHSVEEYMTHVEDYYRTLE
Fut8Dmelanogaster    2 PQPTTRDFLTSGMRN--LGW-ERPIVGVHVRRTDKVGTEAACHSVEEYMTYVEDYYRTLE
Fut8Agambiae         2 PTGETQQMLENGIER--LGF-KKPIVGVHVRRTDKVGTEAAFHGIEEYMTAVDDYYDQLE
Fut8Aaegypti         2 PTGETRQMLENGIEK--LGF-KKPIVGVHVRRTDKVGTEAAFHGIEEYMQAVDDYYDQVE
Fut8Cpipiens         2 PTGETRQMLENGMEK--LGF-RKPIVGVHVRRTDKVGTEAAFHGIEEYMMAVDDYYNQVE
                               :              *  *:::*****:  **  * : **:  .   :
```

Motif I

i7l

```
Fut8Cjaponica          2 RRQ-----GTPLSRRIFIASDDPTVVPEAKLNYP--DYEVLGSTEIAQTAQ-LNNRYTDA
Fut8Celegans             KRQ-----GKPLERRIFIASDDPTVVPEAKNDYP--NYEVYGSTEIAKTAQ-LNNRYTDA
Fut8Cbrenneri          2 KRQ-----GKPLKRRIFIASDDPTVVPEAKNDYP--DYEVFGSTEIAKTAQ-LNNRYTDA
Fut8Cremanei           2 RRQ-----GKPLKRMVFIASDDPTVVPEAQNNYP--EYKVYGSTEIAKTAQ-LNNRYTDA
Fut8Asuum              2 YRI-----GRSIQRRIYIATDDPSVFAEAKEKYS--NYEVYGDVAIADSAQ-TRKRYSSD
Fut8Lloa               2 YRI-----GSSVKRRIYIATDDPEIFDELLMKYP--NYEIYGDPKISKMAQ-VHSRYTIE
Fut8Bmalayi            2 YRT-----RLSVKRRIYIATDDPEVFDEVLMKYP--NYEIYGDPKISNMAQ-VHSRYTVE
Fut8Wbancrofti         2 YRT-----RLSIKRRIYIATDDPEVFDEVLMKYP--NYEIYGDPKISNMAQ-VHSRYTVE
Fut8Tspiralis            LHN-DSFLPAGGKRAVYVATDEPTVFDDLKREYP--NYLFIGSYEVAKSAT-AQKRYTDA
Fut8Turticae           0 IYQQRSGKNDKIERLIYLATDDTNIWREEIKPFEEKGFTFIGDSQIAQSAG-LTSRYSLD   1
Fut8Hmagnipapillata    2 ISK------PFDKKRVFIASDDTSVLIEAKKKYP--EYEFLSDSGVSESAK-VSRRYSDE
Fut8Nvectensis         2 LNQ------AIDSKRVYVASDDPSVFPELQRKYP--DYTFISDQDISKSAG-IASRYSDA
Fut8Moccidentalis        AIH-----GPLDKRRVYIASDDASVLKDAAKLFP--DYEFSGDQDIAKSAA-LKSRYSVE   2
Fut8CintestinalisA     2 QQRNS-QQQEPTRRRVYLATDDPTLLPIAKAKFP--HYTFVSFVHFSRTAGSLFHRYSTD
Fut8Lsalmonis          2 ASL-----GSNVERRVYVASDDPRVLVECRKKFP--HYSFYGDATVARSAA-VSSRYSSD
Fut8SpurpuratusA       2 RRQ------EVPVRKIYLATDDASLLKEAERKYP--KYTFVSDNAISKSAG-LSSRYSED
Fut8Cteleta            2 KSQ------SIEQRAIYLATDDPNLHTEAKTKYP--NYKIIGESEFSKSAG-LSSRYTDN
Fut8SkowalevskiiA      2 RIR------DVPIKRVYLATDEPKLLSEAKQKFP--DYIFVSDNDISRTAG-LGQRYSEE
Fut8Bfloridae          2 KTQ------EVKKKRVYLATDEPNLLTEAQKQYP--EYDFVSDNAISKSAG-LSSRYTDS
Fut8Xtropicalis        2 RRM------QIDKKRVYLATDDPTLLQEAKAKYP--QYEFISDNSISWSAG-LHNRYTEN
Fut8Rnorvegicus        2 RRM------QVDKKRVYLATDDPALLKEAKTKYS--NYEFISDNSISWSAG-LHNRYTEN
Fut8Hsapiens           2 RRM------QVDKKRVYLATDDPSLLKEAKTKYP--NYEFISDNSISWSAG-LHNRYTEN
Fut8Btaurus            2 RRM------QVDKKRVYLATDDPSLLKEAKTKYP--HYEFISDNSISWSAG-LHNRYTEN
Fut8Acarolinensis      2 RRM------HIDKKRVYLATDDPSLLQEAKSKYS--DYEFISDNSISWSAG-LHNRYTEN
Fut8Ggallus            2 RRM------HVDKKRVYLATDDPSLLQEAKSKYP--NYEFISDNSISWSAG-LHNRYTEN
Fut8Mgallopavo         2 RRM------HVDKKRVYLATDDPSLLQEAKSKYP--NYEFISDNSISWSAG-LHNRYTEN
Fut8Tguttata           2 RRM------HVDKKRVYLATDDPSLLQEAKSKYP--NYEFISDNSISWSAG-LHNRYTEN
Fut8DrerioB            2 QRG------HVDKKRVYLATDDPALLQEAKTKYT--DYEFISDNSISWSAS-LHNRYTEN
Fut8Trubipres          2 RRV------HVDKKRVYLATDDPSLLQEAKNKYP--EYEFISDNSISWSAG-LHNRYTEN
Fut8Oniloticus         2 RRA------HVDKKRVYLATDDPSLLQEAKTKYP--DYEFISDNSISWSAG-LHNRYTEN
Fut8Olatipes           2 RRV------RVDKKRVYLATDDPSLLQEAKTKYP--DYEFISDNSISWSAG-LHNRYTEN
Fut8Hmelpomene         0 LSR------EVDARRVYLATDDAAVLEEARKKYP--TYTILGDAGVAQTAA-THRRYSPQ
Fut8Dplexippus         0 MTK------HVDKKRVYLATDDANVLQDARNKYK--EYEFLGDPSIAKTAA-THRRYTPL
Fut8Bmori              0 LTR------PVDVRRVYLATDDANVLDDARQKYP--EYTFLGDPSIAKTAA-THRRYTPL
Fut8Sfrugiperda        0 LTR------PVAVRRVYLATDDANVLEDARSKYP--SYEFLGDASIAKTAA-THRRYTPL
Fut8Msexta               LTR------TVDARRVYLATDDANVLEDARKKYP--EYTFLGDASIAKTAA-THRRYTPL
Fut8Mrotundata           VKP--------DVRRVFLASDDPKVITTAKNRYS--NYEIIGDPEIAEMAS-VAKRYSDS
Fut8Amellifera           TKP--------EVKRVFLASDDPKVITTARNRYS--NYEIIADPEIAETAS-VAKRYSDS
Fut8Bimpatiens           PKP--------EVRRVFLASDDPKVITTARNRYS--NYEIIGDPEIAETAS-VAKRYSDS
Fut8Hsaltator            TKP--------DARRVFLASDDPKVITTARKNYP--NYVIIGDPEIAETAS-VAKRYSDS
FUT8Cfloridanus          TKP--------DVKRVFLASDDPKVITTARKRYT--NYEIIGDPDIAETAS-VAKRYSDV
Fut8Sinvicta             TKP--------DVKRVFLASDDPKVITTARKRYP--NYEIIGDPEIAETAS-VAKRYSDT
Fut8Acephalotes          TKP--------DVRRVFLASDDPKVITTARKRYP--NYEIIGDPEIAETAS-VAKRYSDT
FUT8Aechinatior          TKP--------DVRRVFLASDDPKVITTARKRYP--NYEIIGDPEIAETAS-VAKRYSDT
Fut8Phumanus           2 QNG-----VEINRRRIYLATDDPKVIFEAKNKYP--DYDVIGDPDVAKTAG-MSTRYSDN
Fut8Smaritima          2 LRQ------NVTERRIYLASDDPNVFAEAKRSFP--GFEFIGDPNIAKSAA-MATRYTDS
Fut8Dpulex             2 MKE------KIKVRRVYLASDDPSVLPEAKKKYP--EYEFLGDVSIAKGAA-VATRYTDS
Fut8Apisum             2 LTD------KVDKKRVYLATDEPKLFSEAKRKYP--EYEIVGDEDISKTAS-ISKRYSDQ
Fut8Tcastaneum           LKT------SVDKRRIYLATDDPKVIADAKSKYS--HYEILGDPSISKTAA-ISTRYSDS
Fut8Dmojavensis          ING------SSVVRRIFLASDDARVIVEARKKYP--QYQIVGDPEVARMAA-VSTRYTDT
Fut8Dpseudoobscura       ING------TTVVRRVFLASDDAYVIEEAREKYP--HYQIIGDPEVARMAS-VSTRYTDT
Fut8Dananassae           VNG------TSVVRRIFLASDDALVIEEARRKYP--EYQIIGDPEVARMAS-VSTRYTDT
Fut8Dmelanogaster        VNG------STVARRIFLASDDAQVIEEARRKYP--QYQIIGDPEVARMAS-VSTRYTDT
Fut8Agambiae           0 LTE------KVDKRRVFVASDDPKVIEETKTKYP--HYEVIGDPNVAKMAA-VSTRYTDS
Fut8Aaegypti           0 MVE------AIDKRRVFIASDDPKVIDEAKTKYP--HYEVIGDPDVAKVAA-VSTRYTDS
Fut8Cpipiens           0 MTE------AIDKRRVFIASDDPKVIDEAKSKYP--HYEIIGDPDVAKVAA-VSTRYTDS
                                      : :::*:*:  :      :    : . .   .:  *    **:
```

i7c

Motif II

i8l

```
Fut8Cjaponica          0 SLMGVITDIYILSKVDYLVCTFSSQVCRMGYELRQPTGA-----------DDGSKFHSLD
Fut8Celegans           0 SLMGVITDIYILSKVNYLVCTFSSQVCRMGYELRQPSGA-----------DDGSKFHSLD
Fut8Cbrenneri          0 SLMGVITDIYILSKVDYLVCTFSSQVCRMGYELRQPMGA-----------DDGSKFHSLD
Fut8Cremanei           0 SLMGVITDIYILSKVDYLVCTFSSQVCRMGYELRQPSGA-----------DDGSKFHSLD
Fut8Asuum              2 SLFGVIVDIEMLARCTYLVCTFSSQVCRMGYELMQIRVG-----------DAGSRFHSLD
Fut8Lloa               2 SLIGVVVDIELLSRCIYLVCTFSSQVCRMGYELMQVRFG-----------DAGDRFHSLD
Fut8Bmalayi            2 SLIGIVIDIELLSRCIYLVCTFSSQVCRMGYELMQVRFG-----------DAGDRFHSLD
Fut8Wbancrofti         2 SLIGIVIDIELLSRCIYLVCTFSSQVCRMGYELMQVRFG-----------DAGDRFHSLD
Fut8Tspiralis          0 SLHGIIADVHFLSLCDFLVCTFSSQVCRLAYELMQTRGD-----------DMAYAFRSLD
Fut8Turticae             SLRNVILDIWLLSESDHLVCTFSSQVCRLAYELMQTRGLENNINNEKSRIDWYDAYNSLD   0
Fut8Hmagnipapillata    0 SLHGVISDIEILSDTDLLVCTFSSQVCRMAYEIMNSKHT-----------DASQRFRSLD
Fut8Nvectensis         0 SLHGVIFDIQMLSECDYLVCTFSSQVCRVGYEIMQSKHP-----------DASRKFQSLD
Fut8Moccidentalis      0 SLRGVIADIHMLAQSDYLVCTLSSQVCRVAFELMQLRYP-----------DASQRCQSLD   2
Fut8CintestinalisA     0 SLRGVIADIHLLSKCNYLVCTFSSQVCRVAYELMQTLHP-----------DASNYFHSLD
Fut8Lsalmonis            SLKGVITDIHLLSKADYLVCTFSSQVCRIAYEILQNRFP-----------DGHDRFKSLD
Fut8SpurpuratusA       0 SLRGVIVDIFYLSRSDFLVCTFSSQVCRVAYEMMQDLHP-----------DAAANFRSLD
Fut8Cteleta            0 ALRAIIMDIHFLSHSDYLVCTFSSQVCRVAYEVMQTLHP-----------DASARFKSLD
Fut8SkowalevskiiA      0 SLKGVIADIHLLSESDYLVCTFSSQVCRVAYEIMQTRFP-----------DASTFFHSLD
Fut8Bfloridae          0 ALRGVIIDIHFLAHSDYLVCTFSSQVCRVAYEIMQTLKP-----------DAAANFHSLD
Fut8Xtropicalis        0 SLRGVILDIHFLSQADFLVCTFSSQVCRVAYEIMQTLHP-----------DASAHFHSLD
Fut8Rnorvegicus        0 SLRGVILDIHFLSQADFLVCTFSSQVCRVAYEIMQTLHP-----------DASANFHSLD
Fut8Hsapiens           0 SLRGVILDIHFLSQADFLVCTFSSQVCRVAYEIMQTLHP-----------DASANFHSLD
Fut8Btaurus            0 SLRGVILDIHFLSQADFLVCTFSSQVCRVAYEIMQTLHP-----------DASANFHSLD
Fut8Acarolinensis      0 SLRGVILDIHFLSQADFLVCTFSSQVCRVAYEIMQTLHP-----------DASAFFHSLD
Fut8Ggallus            0 SLRGVILDIHFLSQADFLVCTFSSQVCRVPYEIMQTLHP-----------DASAYFHSLD
Fut8Mgallopavo         0 SLRGVILDIHFLSQADFLVCTFSSQVCRVAYEIMQTLHP-----------DASAYFHSLD
Fut8Tguttata           0 SLRGVILDIHFLSQADFLVCTFSSQVCRVAYEIMQTLHP-----------DASAYFHSLD
Fut8DrerioB            0 SLRGVILDIHFLSQTDFLVCTFSSQVCRVAYEMMQTLHP-----------DASSFFRSLD
Fut8Trubipres          0 SLRGVILDIHFLSQTDFLVCTFSSQVCRVAYELMQMLRP-----------DASSFFYSLD
Fut8Oniloticus         0 SLRGVILDIHFLSQTDFLVCTFSSQVCRVAYEIMQTLHP-----------DASSFFYSLD
Fut8Olatipes           0 SLRGVILDIHFLSQTDFLVCTFSSQVCRVAYEIMQTLHP-----------DASSFFYSLD
Fut8Hmelpomene           SLRGLLRDLHLLSRCDLLVATFSSQVGRVAYEMMQANRP-----------DASQDAVSLD
Fut8Dplexippus         0 SLTGLLVDLHMLAMCDYIVCTFSSQVGRVAYEMMQANRP-----------DASDSFHSLD
Fut8Bmori              0 SLTGLLVDLHMLAMCDYLVCTFSSQVGRVAYEMMQSNRV-----------DASNSFFSLD
Fut8Sfrugiperda        0 SLTGLLVDLHLLAMCDYLVCTFSSQVGRVAYEMMQTNRV-----------DASDSFFSLD
Fut8Msexta               SLTGLLVDLHMLAMCDYIVCTFSSQVGRVAYEMMQTNRV-----------DASNSFFSLD
Fut8Mrotundata           SLQGIIIDIHLLSECDYLVCTFSSQVCRVAYELMQTYYP-----------DAYNRFTSLD
Fut8Amellifera           SLQGIIIDIHLLSECDYLVCTFSSQVCRVAYELMQTYYP-----------DAYNRFASLD
Fut8Bimpatiens           SLQGIIIDIHLLSECDYLVCTFSSQVCRVAYELMQTYYP-----------DAYNRFASLD
Fut8Hsaltator            SLQGIIIDIHLLSECDYLVCTFSSQVCRVAYELMQTFYA-----------DAYNRFTSLD
FUT8Cfloridanus          SLQGIIIDIQLLSECDYLVCTFSSQVCRVAYELMQTYYA-----------DAYDRFSSLD
Fut8Sinvicta             SLQGIIIDIHLLSECDYLVCTFSSQVCRVAYELMQTFYA-----------DAYDRFTSLD
Fut8Acephalotes          SLQGIIIDIHLLSECDYLVCTFSSQVCRVAYELMQTFYA-----------DAYDRFASLD
FUT8Aechinatior          SLQGIIIDIHLLSECDYLVCTFSSQVCRVAYELMQTFYA-----------DAYDRFASLD
Fut8Phumanus             SLFGILLDIHLLSLSDHLVCTFSSQVCRVAYEIMQTYDL-----------DATTHFTSLD
Fut8Smaritima          0 SLRGIILDVHFLSMSDYLVCTFSSQVCRIAYEIMQTVNP-----------NAASNFRSLD
Fut8Dpulex               SLRGILVDIHMLAHSDHLVCTFSSQVCRLAYEIMQTLHP-----------DASSKFKSLD
Fut8Apisum             0 SLSGIITDIHFLSLSDYLVCTFSSQVCRVAYEIMNSLHP-----------DASTLYTSLD
Fut8Tcastaneum         0 SLFGIINDINMLAMSDYLVCTFSSQVCRVAYEIMQTYYP-----------DASSRFRSLD
Fut8Dmojavensis          ALNGIILDIHLLSMSDYLVCTFSSQVCRVAYEIMQTMYP-----------DAAYRFKSLD
Fut8Dpseudoobscura       ALNGIILDIHLLSMSDHLVCTFSSQVCRVAYEIMQTMYP-----------DASHRFKSLD
Fut8Dananassae           ALNGIILDIHLLSMSDHLVCTFSSQVCRVAYEIMQTMYP-----------DAAHRFKSLD
Fut8Dmelanogaster        ALNGIILDIHLLSMSDHLVCTFSSQVCRVAYEIMQTMYP-----------DAAHRFKSLD
Fut8Agambiae             SLNGIILDIHLLSLSDYLVCTFSSQVCRVAYEIMQSMYP-----------DASGRFRSLD
Fut8Aaegypti             SLNGIILDIHLLSLSDYLVCTFSSQVCRVAYEIMQTMYP-----------DASNRFKSLD
Fut8Cpipiens             SLNGIILDIHLLSLSDYLVCTFSSQVCRVAYEIMQTLYP-----------DASGRFKSLD
                         :*  :: *:  *:     :*.*:**** *: :*: :              :      ***
```

Motif III

i9l

```
Fut8Cjaponica           1 DIYYFGGQLAHEVLVIEDHVAQTP-QEIDLKVGDKVGIAGNHWNGYSKGTNRRT--FKEG
Fut8Celegans            1 DIYYFGGQQAHEVIVIEDHIAQNN-KEIDLKVGDKVGIAGNHWNGYSKGTNRQT--YKEG
Fut8Cbrenneri           1 DIYYFGGQQAHEVIVIEDHVALNN-KEIDLKVGDKVGIAGNHWNGYSKGTNRRT--YKEG
Fut8Cremanei            1 DIYYFGGQQAHEVVVIEDHVALNN-KEIDLKVGDKVGIAGNHWDGYSKGTNRRT--YKEG
Fut8Asuum               1 DLYYYGGQLAHEQVVVEAYEAESK-QEISLKVGDVIGVAGNHWDGFSKGVNRRT--GANG
Fut8Lloa                1 DIYYFGGQQAHEQVAVESYRAESG-NEIDLKIGDIIGIAGNHWNGFSMGTNRRT--GKKG
Fut8Bmalayi             1 DIYYFGGQQAHEQVAVESYRAEND-NEIDLKIGDIIGIAGNHWDGFSKGTNRRT--GKKG
Fut8Wbancrofti          1 DIYYFGGQQAHEQVAVESYRAEND-NEIDLKIGDIIGIAGNHWDGFSKGTNRRT--GKKV
Fut8Tspiralis             DVYYFGGQHPHDVTALMDHKPEGP-DEIELKIGDRIGIAGNHWNGYSKGVNRRT--QKSG
Fut8Turticae              DIYYFGGQNAHNQIAILNHDPEPG--QIPMKIGDTIGIAGNHWNGWSKGLNRVN--GKTG   1
Fut8Hmagnipapillata       DIYYFGGQQEHKTRVIWPHTAKGR-SELDLRVGDVIGIAGNHWNGQAKGLLHNL--QKTG   1
Fut8Nvectensis            DIYYFGGQSGHNVRAILPHTAETR-EEIDLEVGDLIGIAGNHWDGYSKGTNHRT--GQMG
Fut8Moccidentalis         DIFYFGGQKDHDEEAIADNLQPDSGGEIAFKKGDIIGIAGNHWDGYSKGINRRT--KQRG
Fut8CintestinalisA        DVYYCGGQTAHEQTAVLDHAPKPGSQEIELRIGDVVVLSGNHWDGYSKGTNRRT--KKTG   0
Fut8Lsalmonis             DIWYFGGQDEHQQEVIIYHGPKTR-DEIELKVGDVIGVAGNHWDGFNKGKNLRN--RRVG
Fut8SpurpuratusA          DIYYYGGQNAHEQTVLYEHKPQGS-DEIEMRPGDSIGVAGNHWDGYSKGSNLRSTPRRAG
Fut8Cteleta               DIYYFGGQNGHSQMAAYPHEPRSGSDEMQLVTGDHVGIAGNHWDGYSKGNNRRT--GKTG
Fut8SkowalevskiiA         DVYYFGGQNAHDEKVLYTHIPRNG-AEIPLELDDAIGIAGNHWDGYSKGHNRRI--NKSG
Fut8Bfloridae             DIYYFGGQGDHNQVAIAKHEPRNQ-QEIYLEQGDELGVAGNHWDGYSKGVNRRT--HKSG
Fut8Xtropicalis           DIYYFGGQNAHNQLAIYPHQPRNA-EEIPLEPGDIIGVAGNHWDGYSKGINRKL--GRTG
Fut8Rnorvegicus           DIYYFGGQNAHNQIAVYPHKPRTD-EEIPMEPGDIIGVAGNHWDGYSKGVNRKL--GKTG
Fut8Hsapiens              DIYYFGGQNAHNQIAIYAHQPRTA-DEIPMEPGDIIGVAGNHWDGYSKGVNRKL--GRTG
Fut8Btaurus               DIYYFGGQNAHNQIAIYPHEPRTA-DEIPMEPGDIIGVAGNHWDGYSKGVNRKL--GRTG
Fut8Acarolinensis         DIYYFGGQNAHNQIAIYAHHPRTA-DEIPMEPGDIIGVAGNHWDGYSKGVNRKL--GKTG
Fut8Ggallus               DIYYFGGQNAHNQIAVYAHHPRTA-DEIPMEPGDIIGVAGNHWDGYSKGINRKL--GKTG
Fut8Mgallopavo            DIYYFGGQNAHNQIAVYAHHPRTA-DEIPMEPGDIIGVAGNHWDGYSKGINRKL--GKTG
Fut8Tguttata              DIYYFGGQNAHNQIAIYAHHPRTA-DEIPMEPGDIIGVAGNHWDGYSKGINRKL--GRTG
Fut8DrerioB               DIYYFGGQNAHNQIAIYPHQPRTA-EDIPLDPGDVIGVAGNHWDGNSKGINRKL--GRTG
Fut8Trubipres             DIYYFGGQNAHNQIAIYPHQPRHG-DDIPLEPGDVVGVAGNHWDGYSKGVNRKL--GRTG
Fut8Oniloticus            DIYYFGGQNAHNQIAVYPHQPRNS-EDIPLEPGDVIGVAGNHWDGYSKGINRKL--GRTG
Fut8Olatipes              DIYYFGGQNAHNQIAIYPHQPRNS-EDIPLEPGDVIGVAGNHWDGYSKGINRKL--GRTG
Fut8Hmelpomene          0 DIYYFGGQNAHDRRAVMPHTALDH-HQLDLQMGDLIGIAGNHWNGFGRGTNKRT--NLNG   0
Fut8Dplexippus          0 DIYYFGGQNAHDRRALMNHEA-GG-QEISFQAGDLIGIAGNHWNGFGRGTNKRT--NLNG   0
Fut8Bmori               0 DIYYFGGQNAHDRVAIMQNHGGKN-EDISFEVGDKIGVAGNHWNGYGRGTNKRT--NMNG   0
Fut8Sfrugiperda         0 DIYYFGGQNAHDRVAVMPNDG-AY-QDISFQVGDLIGIAGNHWNGYGRGTNKRT--NMNG   0
Fut8Msexta                DIYYFGGQNAHDRVAVMSNTGGKS-QDISFEAGDRIGIAGNHWNGYGRGTNKRT--NVNG
Fut8Mrotundata            DIYYYGGQNPHPHKVILDHQPRRS-GEIELKVGDLVEVFGNHWDGYSKGYNTRT--SMTG
Fut8Amellifera            DIYYYGGQNSHPHQVILDHKPRKD-GEIELKVGDLIEVLGNHWDGYSKGYNIRT--SMTG
Fut8Bimpatiens            DIYYYGGQNPHPHQAILDHKPRKE-GEIELKVGDLIEVFGNHWDGYSKGYNTRT--SMTG
Fut8Hsaltator             DIYYYGGQNPHPHVAIIDHKPRKN-GELELKAGDLIEVYGNHWDGFSKGYNSRT--SMTG
FUT8Cfloridanus           DIYYYGGQNPHPHVAILDHKPRKN-GELEMKIGDLIEVYGNHWDGFSKGYNTRT--SMTG
Fut8Sinvicta              DIYYYGGQNPHPHVAILDHKPRKN-GELELKTGDLIDVFGNHWDGYSKGYNTRT--SMTG
Fut8Acephalotes           DIYYYGGQNPHPHVAILDHKPRKN-GELELKVGDLIDVFGNHWDGFSKGYNTRT--SMTG
FUT8Aechinatior           DIYYYGGQNPHPHVAILGHKPRKN-GELELKIGDLIDVFGNHWDGFSKGYNTRT--SMTG
Fut8Phumanus            0 DVYYYGGQNAHSSVAVLPHEPMNS-NEIELKVGDKIIVAGNHWDGYSKGRNLRT--NKEG
Fut8Smaritima             DIYYFGGQNAHNHRVIYSHESQAS-NELELMAGDVIGIAGNHWDGYSKGINRRT--KQQG
Fut8Dpulex                DIYYYGGQGPHQQIAMYSHKAHRP-GEISIEVGDVLGIAGNHWDGYSKGTNERT--KQSG
Fut8Apisum                DIYYYGGQKRRLHVAVLPHKANGP-HEMNLMVGDEIAVAGNHWDGYSKGTNLRT--KESG
Fut8Tcastaneum            DVYYYGGQNPHNTIAVLSHEPRKN-GEMSVVAGDLIGVAGNHWNGFSKGRNLRT--NQIA
Fut8Dmojavensis           DIYYYGGQNPHNRRAVIPHKPRSH-EDLQLKVGDLVSVAGNHWDGNSKGKNTRT--NQGG
Fut8Dpseudoobscura        DIYYYGGQNAHNRRVVIAHSPRTH-EDLQLRVDDLVSVAGNHWDGNSKGKNTRT--NQGG
Fut8Dananassae            DIYYYGGQNAHNRRVVIAHKPRSH-EDLQLRVGDLVSVAGNHWDGNSKGKNTRT--NQGG
Fut8Dmelanogaster         DIYYYGGQNAHNRRVVIAHKPRTH-EDLQLRVGDLVSVAGNHWDGNSKGKNTRT--NQGG
Fut8Agambiae              DIYYYGGQNSHNREVVLPHEPKNH-DEIQIRPGDLVGVAGNHWNGYSKGKNLRT--NQVG
Fut8Aaegypti              DIYYYGGQNAHNREVVLPHNPKNH-DEIHMRKGDLIGVAGNHWNGYSKGKNVRT--GQVG
Fut8Cpipiens              DIYYYGGQNAHNREVVLAHNPKNH-DEIHMRKGDLVGVAGNHWNGYSKGKNSRT--SQVG
                          *::* ***  :   .           :: .   * : : ****:*   *
```

i*10*l i*11*l

```
Fut8Cjaponica              VFPSYKVENDWRTFEFSALLD-------------
Fut8Celegans               VFPSYKVVNDWRKFKFEALLD-------------
Fut8Cbrenneri              VFPSYKVVNDWRQFNFEALLD-------------
Fut8Cremanei               VFPSYKVVNDWRQFNFEALLD-------------
Fut8Asuum                  LYPSYKVREKWIVVPFP-----------------
Fut8Lloa                   LYPSYKAREKYIIVDFP-----------------
Fut8Bmalayi                LYPSYKAREKYITLDFP-----------------
Fut8Wbancrofti             FIHLTKFVKS----ISP-----------------
Fut8Tspiralis              FYPSYKVEEAWNLAETPYDW-SEILK--------
Fut8Turticae               LYPGFKADEKIDIAKFEAFDNIDT*---------
Fut8Hmagnipapillata        LFPAYKIEDIHDVEEFPIPKE-------------
Fut8Nvectensis             LYPSYKVEEVMETAQFPTFDDL------------
Fut8Moccidentalis          LYPSFKTKEHVDAVDFGDFIL-------------
Fut8CintestinalisA         LYPSYKVNEKIPLLKYLTYPEADSYKGEGNT---
Fut8Lsalmonis              LYPEYKTKEKFRIVEFRGLRGSEQS*--------
Fut8SpurpuratusA           LYPSYKVEDKTNIAKMPTYPEAENFQL-------
Fut8Cteleta                LFPSYKMVEDVEIVDFPTYPEATPQRGGGSR---
Fut8SkowalevskiiA          LYPSYKTEDKPNIAKMPTYPEADKVEL-------
Fut8Bfloridae              LYPSYKVEEKIELVKYPTYPEVEESS--------
Fut8Xtropicalis            LYPSYKVKEKIETVKYPTYQEAEK----------
Fut8Rnorvegicus            LYPSYKVREKIETVKYPTYPEAEK----------
Fut8Hsapiens               LYPSYKVREKIETVKYPTYPEAEK----------
Fut8Btaurus                LYPSYKVREKIETVKYPTYPEAEK----------
Fut8Acarolinensis          LYPSYKVKEKIETVKYPTYPEADK----------
Fut8Ggallus                LYPSYKVKEKIETVKYPTYPEAEK----------
Fut8Mgallopavo             LYPSYKVKEKIETVKYPTYPEAEK----------
Fut8Tguttata               LYPSYKVKEKIETVKYPTYPEADK----------
Fut8DrerioB                LYPSYKVKEKIETVKYPTYPEADKMLSS------
Fut8Trubipres              LYPSYKVKEKIETVKYPTYPEADKLLTAQK----
Fut8Oniloticus             LYPSYKVREKIETVKYPMYPEADKLLNSQNK---
Fut8Olatipes               LYPSYKVKEKIETVKYPLYP--------------
Fut8Hmelpomene             LVPWYKTADHLVLYPFPEYRHASTDS--------
Fut8Dplexippus             LIPWYKTADHLVLYPFPEYKHLQ--TETRQKDL-
Fut8Bmori                  LIPWYKTADHLVLYPFPEYKQVPIYSETSQKNV-
Fut8Sfrugiperda            LIPWYKTADHLVLYPFPEYKQVPLYTDTRQKDL-
Fut8Msexta                 LIPWYKTADHLVLYPFPEYKQVPQYSDTRQKNL-
Fut8Mrotundata             LFPSFKVKNPVDAVDFPKYPNVPLNENKNE----
Fut8Amellifera             LFPSFKVKNPVDAVDFPKYSNVPLRENKNE----
Fut8Bimpatiens             LFPSFKVKNPVDAVDFPKYPYVPLQENRNE----
Fut8Hsaltator              LFPSYKVKNRVAAVDFPKYPSVPLLENKID----
FUT8Cfloridanus            LFPSFKVKNPVDAVDFPKYSNVLLVEDKTD----
Fut8Sinvicta               LFPSFKVKNPVDAVDFPKYSNISPLENKDD----
Fut8Acephalotes            LFPSFKVKNPIDAVDFPKYSNIPLLENKED----
FUT8Aechinatior            LFPSFKVKNPVDAVDFPKYSNIPLLENKED----
Fut8Phumanus             0 LYPSFKVQNRVKVVDFPVNDGGLTF---------
Fut8Smaritima              LYPSFKATNKLQIADFPTYPEVKQNELSSSR---
Fut8Dpulex                 LYPSFKAVDKYNIIDFPPYSEVAVAA--------
Fut8Apisum               0 LYPTFKVSPKIETAPFASYPDVTLSTNELQEQKR
Fut8Tcastaneum             LYPSFKVKDKIETAKFPTYAEVDKHENDDQQIS-
Fut8Dmojavensis            LFPSFKVVDKVETAKLPVYPGV*-----------
Fut8Dpseudoobscura         LFPSFKVEEKIDTAKLPLYPGV------------
Fut8Dananassae             LFPSFKVEEKVDTAKLPLYPGV------------
Fut8Dmelanogaster          LFPSFKVEEKVDTAKLPLYAGI------------
Fut8Agambiae               LFPSFKVNDKIEIVELPKYPYVK-----------
Fut8Aaegypti               LFPSFKVNDKIEVVELPTYPNVK-----------
Fut8Cpipiens               LFPSFKVNDKIEVVDLPKYPKVK-----------
                           .    *
```
