# Supplementary figures and images for: The α1,6-Fucosyltransferase Gene (fut8) from the Sf9 Lepidopteran Insect Cell Line: Insights into fut8 Evolution
Source: PLoS One. 2014 Oct 21;9(10):e110422. doi: 10.1371/journal.pone.0110422 (PMC4204859; doi:10.1371/journal.pone.0110422)

Figure S2

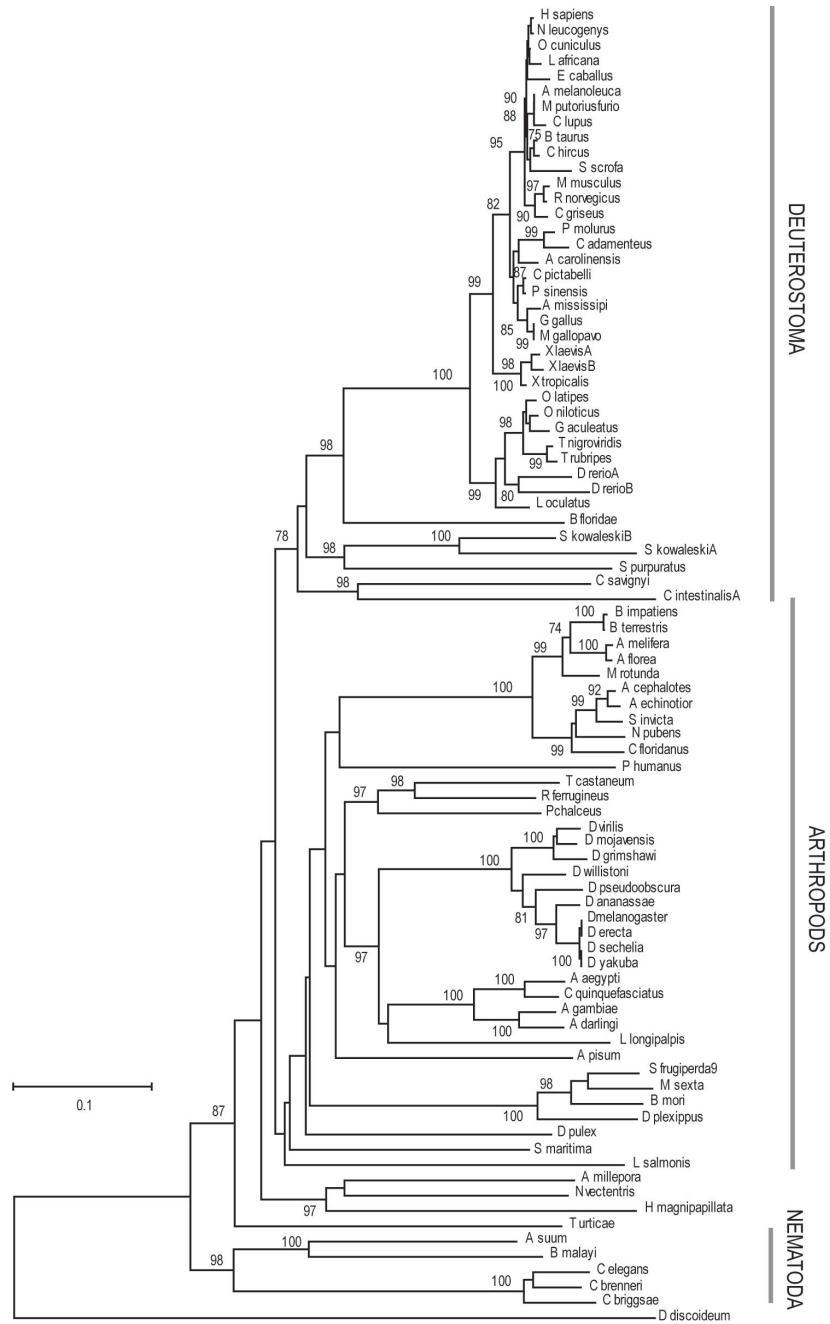

Supplement: Figure S2 — The evolutionary history was inferred using the Neighbor-Joining method [68] and 86 amino acid sequences aligned with MAFFT (EBI). All positions containing gaps and missing data were eliminated. The final dataset contained 461 positions selected in 17 blocks by GBlocks [66] (58% of the original 785 positions). The Dictyostelium discoideum sequence was used as outgroup. The optimal tree with a branch length sum = 6.93435487 is shown. The percentage of replicated trees (>75%) in which the associated taxa clustered together in the bootstrap test (1050 replicates) is shown next to the branches. The tree is drawn to scale, with branch lengths (next to the branches) in the same units as those of the evolutionary distances used to infer the phylogenetic tree. The evolutionary distances were computed using the p-distance method [69] and refers to the number of amino acid differences per site. (PDF) [file pone.0110422.s002.pdf]
