# Supplementary material for: The α1,6-Fucosyltransferase Gene (fut8) from the Sf9 Lepidopteran Insect Cell Line: Insights into fut8 Evolution
Source: PLoS One. 2014 Oct 21;9(10):e110422. doi: 10.1371/journal.pone.0110422 (PMC4204859; doi:10.1371/journal.pone.0110422)
Supplement: Table S1 — Primers used in this study. (PDF) [file pone.0110422.s003.pdf]

**Table S1**

| Primer | | Sequence |
|---|---|---|
| **Cloning of cDNA** | | |
| ForFut1deg | | AACAARGGYTGTGGYTAYGGCTGYCARCTSCAYCATGTG |
| BacFut2deg | | GTGSGCATTYTGRCCNCCRWARTAGTAKATGTCRTCYAR |
| For3'RACEFut8 | | CAGAGTGGCGTACGAGATGATGCAGCA |
| Bac5'RACEFut8 | | GTACCTCCAGCCTTTGGAGTTGAGGATCAGG |
| **Cloning of introns** | | |
| . Intron 1 | UVSF1 | GCGGCAACAGTGAGGAGTCGCTACAAGGATGCCGCCGCG |
| | BacFutEco | CCTCTCCTGGAATTCTGCTACCTCATC |
| . Intron 2 | ForFutEco | GATGAGGTAGCAGAATTCCAGGAGAGG |
| | Fut8B5 | GGGTCCCTGAACCCCGTCTAGGAACAGGGACCC |
| . Intron 3 | Forint10 | GGTAACCTGAAGGACATAGACACAGGGTCCC |
| | B22 | CTGCCTCCACTCATTGTACCCGTCCAGC |
| . Intron 4 | Forint2 | CGTGCAACGACGACTGCACTATCTACAGAACC |
| | Bac5'RACEFut8 | GTACCTCCAGCCTTTGGAGTTGAGGATCAGG |
| . Introns 5 and 6 | ForFut1 | AATAAGGGTTGCGGCTTCGGTTGCCAGCTACATCACATA |
| | Bac21 | GATAGCTTTCTGCATCGCCTGGCGCGGC |
| . Intron 7 | Fut8F6 | GGCCAGATGCTGAAATACATTCTGAAGCCGCGCCAGGCG |
| | Fut8B9 | CGTGGATGTGGTGGAACGCCGCCTCCGTGCCC |
| . Intron 8 | Fut8F11 | GGACTACTACGACCAGCTCGAGCTCACCCGCCCCG |
| | Fut8B13 | GGCCGCCGTCTTGGCGATGGACGCGTCGCCC |
| . Introns 9 and 10 | B13F | GGGCGACGCGTCCATCGCCAAGACGGCGGCC |
| | B19 | GCCTCGACCGTAACCGTTCCAGTGGTTGCCCGCG |
| . Intron 11 | Fut8F19 | GGTTGGCGATTTAATAGGCATCGCGGGC |
| | BacStop | GCAAGGATCCTTATAAGTCTTTCTGTCGCGTGTCT |
| **Synthesis of Southern probe** | | |
| B24 | | CCTTTCACCAATTTGGTCC |
| Forint10 | | GGTAACCTGAAGGACATAGACACAGGGTCCC |
