# Supplementary material for: The α1,6-Fucosyltransferase Gene (fut8) from the Sf9 Lepidopteran Insect Cell Line: Insights into fut8 Evolution
Source: PLoS One. 2014 Oct 21;9(10):e110422. doi: 10.1371/journal.pone.0110422 (PMC4204859; doi:10.1371/journal.pone.0110422)
Supplement: Table S2 — Nomenclature, name and GenBank accession number of the fut8 gene sequences used in this study. (*) Nucleotide sequence data available in the Third Party Annotation Section of the DDBJ/EMBL/GenBank databases. (**) Ensembl accession number. (PDF) [file pone.0110422.s004.pdf]

Table S2

Species	Protein length (aa)	GenBank Accession Number
Acromyrmex echinatior	590	EGI57854
Acropora millepora	597	JR981089
Acyrthosiphon pisum	567	XM_001947060
Aedes aegypti	573	XM_001658942
Ailuropoda melanoleuca	575	XM_002914155
Alligator mississippiensis	576	XP_006266233
Anolis carolinensis	576	XM_003214611
Anopheles darlingi	595	EFR26214
Anopheles gambiae	586	XM_321174
Apis florea	590	XM_003698480
Apis mellifera	590	XM_624898
Ascaris suum	570	JI169392
Atta cephalotes	597	BK008808*
Bombus impatiens	590	XM_003485945
Bombus terrestris	590	XM_003398241
Bombyx mori	562	XM_004928104
Bos taurus	575	BC104555
Branchiostoma floridae	553	XM_002613411
Brugia malayi	591	XP_001895912
Caenorhabditis brenneri	560	EGT47213
Caenorhabditis briggsae	589	XM_002636779
Caenorhabditis elegans	559	NM_072154
Caenorhabditis japonica	580	CJA11459a**
Caenorhabditis remanei	560	XM_003112605
Camponotus floridanus	590	EFN69737
Canis lupus familiaris	575	AJ830717
Capitella teleta	589	ELU04531
Capra hircus	575	JO595826
Chrysemys picta belli	576	BK008807*
Ciona intestinalis A	580	XM_002129700
Ciona savignyi	535	BK008812*
Cricetulus griseus	575	XM_003501735
Crotalus adamanteus	576	JU174603
Culex quinquefasciatus pipiens	573	XM_001849824
Danaus plexippus	557	EHJ67895
Danio rerio A	580	AJ781407
Danio rerio B	579	XM_002666977
Daphnia pulex	585	EFX69666
Dictyostelium discoideum	603	XM_635964
Drosophila ananassae	618	XM_001964192
Drosophila erecta	619	XM_001977053
Drosophila grimshawi	628	XM_001992034
Drosophila melanogaster	619	AF441264
Drosophila mojavensis	627	XM_002010997
Drosophila pseudoobscura	625	AJ830720
Drosophila sechelia	619	XM_002044075
Drosophila virilis	625	XM_002058101
Drosophila willistoni	644	XM_002064234
Drosophila yakuba	619	AJ830719
Equus caballus	575	XM_001499525
Gallus gallus	575	NM_001004766
Gasterosteus aculeatus	583	ENSGACT00000011244**
Harpagnathos saltator	592	BK008805*
Heliconius melpomene melpomene	551	BK008802*
Homo sapiens	575	AJ536056
Hydra magnipapillata	575	XM_002161792
Lepeophtheirus salmonis	572	BK008813*
Lepisosteus oculatus	580	BK008809*

Loa loa	550	XM_003142262
Loxodonta africana	575	XM_003408717
Lutzomyia longipalpis	>473	BK008810*
Manduca sexta	564	JO831253
Megachile rotundata	590	XM_003699654
Meleagris gallopavo	575	XM_003206402
Metaseiulus occidentalis	550	XM_003740372
Mus musculus	575	NM_016893
Mustela putorius furo	583	XM_004738951
Nematostella vectensis	554	XM_001635608
Nomascus leucogenys	575	XM_003267827
Nylanderia pubens	590	JP776325
Oreochromis niloticus	583	XM_003446349
Oryctolagus cuniculus	575	XM_002719524
Oryzias latipes	572	XM_004083551
Pediculus humanus corporis	608	XM_002428546
Pelodiscus sinensis	575	XP_006113475
Pogonus chalceus	592	JU424712
Python molurus	576	BK008806*
Rattus norvegicus	575	NM_001002289
Rhynchophorus ferrugineus	594	JR486628
Saccoglossus kowalevskii A	530	XM_002736283
Saccoglossus kowalevskii B	587	XM_002735425
Solenopsis invicta	590	EFZ14951
Spodoptera frugiperda	561	KC538901
Strigamia maritima	578	BK008814*
Strongylocentrotus purpuratus	555	XM_001197873
Sus scrofa	575	D86723
Taeniopygia guttata	575	XM_002199852
Takifugu rubripes	582	AJ781408
Tetranychus urticae	614	BK008811*
Tetraodon nigroviridis	582	CAH03677
Tribolium castaneum	592	XM_964018
Trichinella spiralis	545	XM_003375058
Trichoplax adherens	>402	XM_002108606
Wuchereria bancrofti	582	EJW88904
Xenopus laevis A	578	AJ514872
Xenopus laevis B	578	BC125984
Xenopus tropicalis	578	BC067957
